# Proteomic Profiling of Plant and Pathogen Interaction on the Leaf Epidermis

**DOI:** 10.3390/ijms232012171

**Published:** 2022-10-12

**Authors:** Yasir Sidiq, Daisuke Tamaoki, Takumi Nishiuchi

**Affiliations:** 1 Division of Life Science, Graduate School of Natural Science and Technology, Kanazawa University, Kanazawa 920-1192, Japan; 2 Biology Education Department, Faculty of Teacher Training and Education, Universitas Muhammadiyah Surakarta, Kartasura 57162, Indonesia; 3 Faculty of Science, Academic Assembly, University of Toyama, Toyama 930-8555, Japan; 4 Division of Integrated Omics Research, Bioscience Core Facility, Research Center for Experimental Modeling of Human Disease, Kanazawa University, Kanazawa 920-8640, Japan

**Keywords:** shotgun proteomics, plant–pathogen interaction, plant immunity, effector, secretory proteins, label-free quantification, fungal pathogen, leaf epidermis

## Abstract

The plant epidermis is the first line of plant defense against pathogen invasion, and likely contains important regulatory proteins related to the plant–pathogen interaction. This study aims to identify the candidates of these regulatory proteins expressed in the plant epidermis. We performed comparative proteomic studies to identify rapidly and locally expressed proteins in the leaf epidermis inoculated with fungal phytopathogen. The conidia solutions were dropped onto the Arabidopsis leaf surface, and then, we collected the epidermal tissues from inoculated and mock-treated leaves at 4 and 24 hpi. The label-free quantification methods showed that expressions of Arabidopsis proteins, which are related to defense signals, such as BAK1, MKK5, receptor-like protein kinases, transcription factors, and stomatal functions, were rapidly induced in the epidermal tissues of inoculated leaves. In contrast, most of them were not differentially regulated by fugal inoculation in the whole leaves. These findings clearly indicate that epidermal proteomics can monitor locally expressed proteins in inoculated areas of plant tissues. We also identified the 61 fungal proteins, including effector-like proteins specifically expressed on the Arabidopsis epidermis. Our new findings suggested that epidermal proteomics is useful for understanding the local expressions of plant and fungal proteins related to their interactions.

## 1. Introduction

Plants recognize pathogen-derived molecular patterns through their receptors and activate the plant immune response. In the case of pathogenic fungi, their conidia adhere to the surface of host plant tissues and germinate through recognition of the plant-derived molecules [1,2]. During these plant–pathogen interactions, protein expression at the plant surface determines whether the plant successfully defends against the pathogen attacks. Pathogens produce secretory proteins, such as effector-like proteins [3], whereas plants are thought to secrete suppressors of pathogenic effectors and antifungal proteins, including pathogenesis-related proteins [4]. It is known that plant secretory antifungal proteins interact with fungal effector-like proteins [5]. Both plants and pathogens rapidly produce these proteins on the surface of the plant attacked by pathogens. However, the local response of protein expression during plant–pathogen interaction remains unknown.

Some proteomic studies using plant epidermal tissues have been reported. Kaspar analyzed UV-B-induced protein expression in the epidermal tissues of barley seedlings [6]. Proteome analysis showed that many defense-related proteins were expressed in potato tuber skins [7]. Schneider reported a proteomic study on the leaf epidermis of *Noccaea caerulescens* during Zinc hyperaccumulation [8]. These studies indicated the importance of protein expressions in the epidermal tissues in response to environmental stimuli. Furthermore, proteomic studies have been conducted using trichomes and guard cells in the epidermal tissues [9,10]. Thus, epidermal proteomics is likely useful for profiling protein expression in plant–pathogen interactions. The simple preparation of epidermal tissues was reported in Arabidopsis leaves using adhesive tapes [11]. Although we also tried this method, we could not prepare the epidermal tissues from Arabidopsis leaves in a short time. Therefore, we peeled the epidermis from Arabidopsis leaves using forceps and collected epidermal tissues within 5 min.

It is known that fungal pathogens enter plant tissues through the stomata of the plant epidermis. *Fusarium* species such as *F. graminearum*, which causes fusarium head blight (FHB) disease in cereal crops, can gain entry to host tissues via the stomata of the lemma in wheat spikes. FHB causes not only yield but also quality loss, which means trichothecene mycotoxin contamination in wheat and barley grains. *F. graminearum* can infect both the leaves and flowers of dicotyledonous Arabidopsis plants [5,12]. It has been reported that macroconidia of *F. graminearum* started to germinate 3 to 4 h after inoculation on the complete medium. Seong et al. showed that macroconidia from binucleate cells were germinated 3 h after inoculation on the plant surface [13]. Therefore, it was expected that both plant and fungal proteins would be rapidly expressed on the plant surface in response to fungal inoculation.

In this study, we tried to monitor proteins expressed on the leaf surfaces during plant–pathogen interaction. For this purpose, we first established *Fusarium* inoculation systems on the Arabidopsis leaf surface. Then, we confirmed early events, such as the germination of conidia and entry of hyphae into host tissues. The Japanese H3 strain *F. graminearum* did not form an appressorium to penetrate the host plant tissues through stomata. Then, we prepared the epidermal tissues from Arabidopsis leaves inoculated with *F. graminearum*. We identified differentially expressed plant and fungal proteins in the epidermal tissues of inoculated leaves using shotgun proteomics with a label-free quantification method.

## 2. Results

### 2.1. Early Events of the Invasion of F. graminearum on the Arabidopsis Leaf Surface

**Figure 1:**
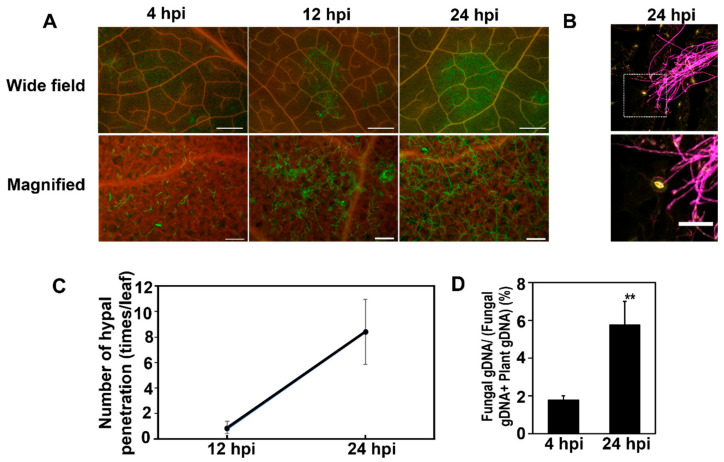
Analysis of *F. graminearum* colonization on the surface of Arabidopsis leaves at 4, 12, and 24 h post-inoculation (hpi). (**A**) Visualization of fungal hyphae stained with WGA-Alexa488 (green) under a fluorescence microscope at 4 and 24 hpi. Red represents chlorophyll autofluorescence. Bars of wide field = 1000 μm and bars of high magnification = 100 μm. (**B**) Mycelium stained by WGA-Alexa 488, and stomata cells stained by PI. Yellow fluorescence is stomata, whereas the purple one is mycelium. Bar = 5 µm. (**C**) Rate of entry of fungal hyphae into leaf tissues. Data represent mean ± standard error. (**D**) Quantification of fungal genomic DNA (gDNA) using quantitative PCR (qPCR). Fungal gDNA was quantified in the leaf epidermis at 4 and 24 hpi. Error bars represent standard deviation (*n* = 3), and asterisks indicate significant difference (** *p* < 0.001; Student’s *t*-test).

**Figure 2:**
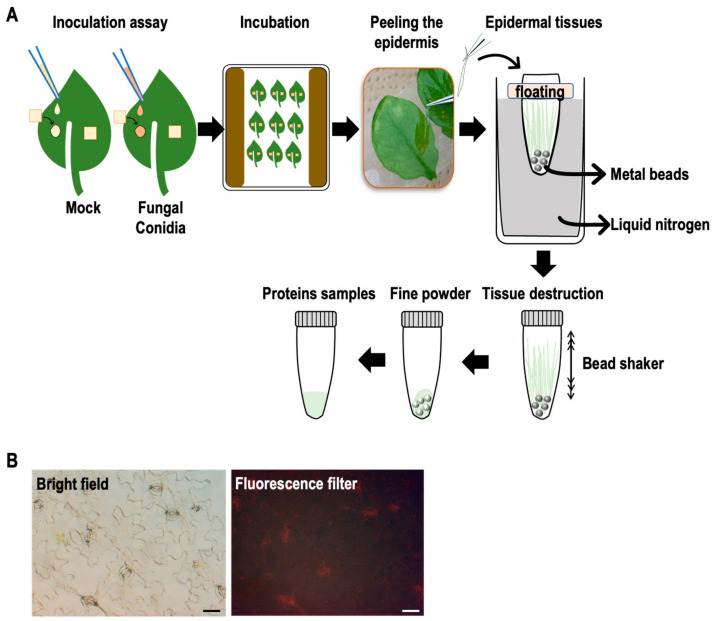
Illustration of leaf inoculation, epidermal peel preparation, and protein extraction. (**A**) The leaf epidermis was peeled rapidly within 3 min per leaf (inoculated and control) and subsequently frozen in liquid nitrogen. Whole inoculated and control leaf samples were also prepared in this experiment. (**B**) Examination of the epidermal peel under a fluorescence microscope. Scale bars represent 50 μm. Red in the image represents chlorophyll autofluorescence.

Inoculation systems were established to monitor proteins expressed on the surface of Arabidopsis leaves after the inoculation of the *F. graminearum*. For this purpose, we inoculated Arabidopsis leaves with a conidial suspension of *F. graminearum* and covered the inoculated conidia solutions with a small piece of nylon mesh to keep humidity high. To observe the conidia and germinated mycelium of inoculated leaves, they were stained with the WGA-Alexa fluor 488 conjugate. The conidia germination was detected within 4 h post-inoculation (hpi), and the significant extension of mycelium occurred by 24 hpi (Figure 1A and Figure 2B). In addition, the entry of *F. graminearum* hyphae into the leaf tissues occurred through the open stomata by 24 hpi (Figure 1C). Correspondingly, the fungal gDNA content was 1.9% at 4 hpi and then increased to 5.8% at 24 hpi (Figure 1D). These results suggested that molecular interaction between Arabidopsis and *Fusarium* activated on the leaf surfaces within 24 dpi.

### 2.2. Many Proteins Were Rapidly Induced on the Leaf Epidermis via Inoculation of the Fungal Pathogen

**Figure 3:**
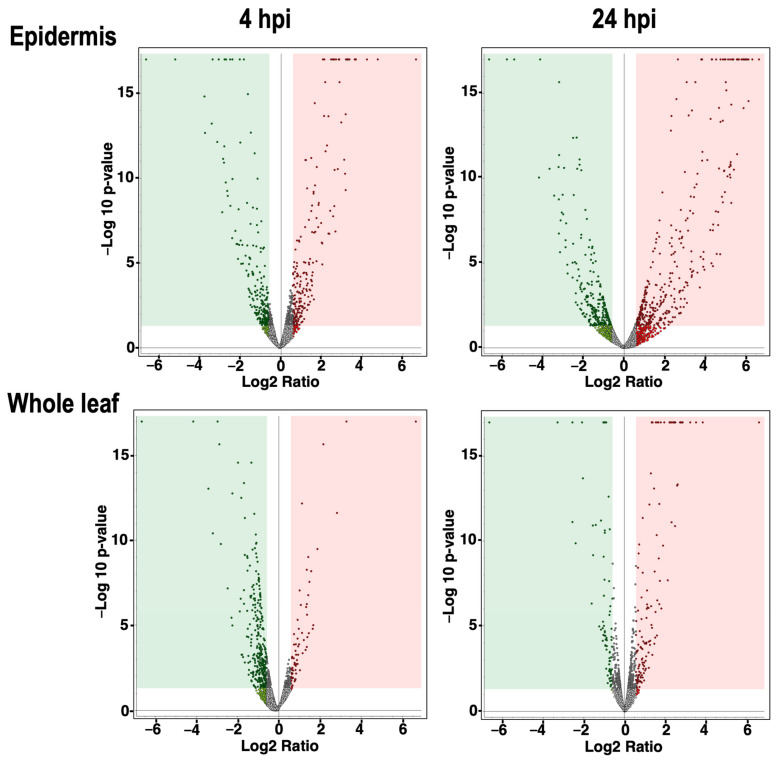
Volcano plots illustrate the spread of quantified proteins in the leaf epidermis inoculated by *F. graminearum*. The plots show the distributed proteins at 4 hpi and 24 hpi of the leaf epidermis. Expression pattern of proteins in the whole leaf at 4 hpi and 24 hpi was also measured. The vertical axis is −log10 of the p-value (cutoff value 0.05), whereas the horizontal axis is log fold change (cutoff value 1.5). The dots indicate all the identified proteins in this experiment. Red and green square areas represent the significantly up-regulated and down-regulated proteins, respectively.

To monitor the expressed proteins on the inoculated Arabidopsis leaf surfaces, we peeled the epidermis of Arabidopsis leaves dropped with or without conidia of *F. graminearum* at 4 and 24 hpi and immediately transferred them into sampling tubes with the floater on liquid nitrogen. Whole-leaf samples were also prepared without peeling the epidermis similarly. The collected leaf epidermis and whole-leaf samples were crushed using the beads shaker. As shown in Figure 2 and Supplementary Figure S1, the proteins were prepared from the collected epidermis and whole leaves with or without inoculation (four replicates per treatment). The prepared proteins were subjected to comparative shotgun proteomic studies. The amounts of expressed proteins were quantified by the label-free quantification method. The results were briefly evaluated by volcano plots (cutoff values: Abundance Ratio > 1.5, *p*-value < 0.05) (Figure 3); the dots in red and green areas represent up- and down-regulated proteins, respectively. The number of differentially regulated proteins during the pathogen challenge was greater in epidermal tissues than in whole leaves (Figure 3).

The number of up-regulated proteins during pathogen challenge in the epidermal tissues were 192 and 280 at 4 and 24 hpi, respectively. Those numbers decreased to 77 and 127 proteins at 4 and 24 hpi, respectively (Supplementary Figure S2; Tables S1 and S2). As stated above, conidia of *F. gaminearum* were germinated within 4 hpi; many plant proteins were already induced on the surface of leaves. These results indicated that epidermal proteomics is useful for identifying early and local responsive proteins upon pathogen challenge. Then, we performed gene ontology (GO) enrichment analysis to profile up-regulated proteins in the epidermis by fungal inoculation. As shown in Table 1, translation, peptide, and amide biosynthesis processes, defense response to fungus, and incompatible interaction were enriched at 4 hpi.

On the other hand, different terms of biological process categories were enriched at 24 hpi. Proteins with functions in organonitrogen compound biosynthetic process, protein stabilization, and membrane permeability were enriched (Table 2).

### 2.3. Many Up-Regulated Epidermal Proteins Were Related to Plant Defense Response

**Figure 4:**
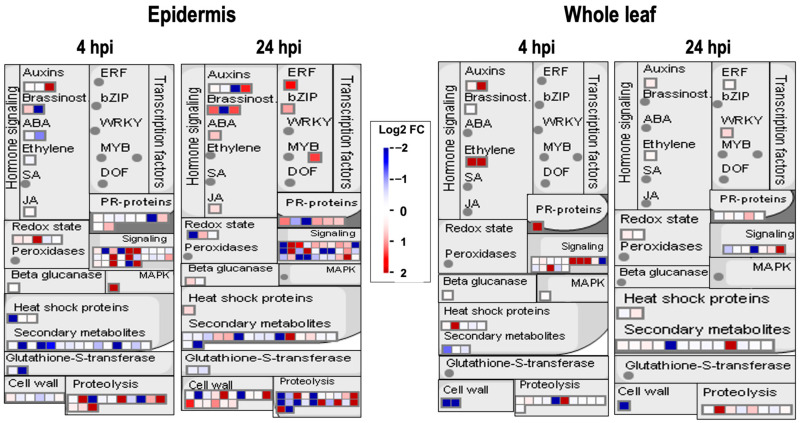
Mapping of proteins identified in the leaf epidermis and whole leaf, based on MapMan bin codes of the Isoform Model TAIR10 database (August 2012). Proteins identified in the leaf epidermis and whole leaf were listed with log2 FC values. Then, protein lists were mapped to the MapMan database.

We analyzed proteomic data using MapMan to profile further up-regulated proteins in the inoculated epidermis [14]. The mapped proteins were presented as diagrams of metabolic, signaling pathways or other events [14]. We show the results of MapMan based on the category of pathogen or pest attack (Figure 4). Identified proteins with log2 values of Fold Change (=Abundance Ratio, inoculated/mock) were mapped to this category. Figure 4 shows that the numbers of mapped proteins in the epidermis at 4 and 24 hpi were greater than in the whole leaves. These proteins are already expressed in the leaf epidermis without pathogen challenge. Furthermore, these epidermal proteins at 4 and 24 hpi were differentially regulated by the inoculation of *F. graminearum* (Figure 4). In contrast, fungal inoculation did not largely affect protein expressions in whole leaves.

Among the 26 signaling-related plant proteins in the epidermis at 4 hpi, 8 were up-regulated by fungal inoculation. Six of these eight proteins were classified into protein kinases, including MKK5. It has been reported that MKK5 was involved in stomatal response and root growth through the MAPKKK20–MKK5–MPK6 cascade [15]. In addition, MKK5 is reported as a key regulator of stomatal development and patterning [16]. In addition, MKK5 is involved in the plant’s innate immune response against bacterial flagellin (flg22) through the MKK1–MKK4/MKK5–MPK3/MPK6 cascade. MKK5 acts downstream of the flg22 receptor kinase (FLS2) and upstream of the WRKY29 transcription factor, which leads to the activation of plant disease resistance [17]. Recently, we revealed that MKK5 positively regulated plant immunity against PstDC3000 and *Fusarium Sporotrichioides* through the MAPKKK δ-1 (MKD1)-MKK1/MKK5-MPK3/MPK6 pathway [18]. The receptor-like protein kinase, THESEUS 1 (THE1), was important for maintaining cell wall integrity in plants.

Microarray analysis suggested that THE1 was involved in the expression of genes encoding transcription factors, defense-related proteins, and inhibitors of fungal enzymes [19]. Some up-regulated epidermal proteins at 4 hpi were mapped in the hormone signaling pathway. Among them, the JA precursor biosynthetic enzyme, lipoxygenase 3 (LOX3) protein, was up-regulated at 4 hpi. Interestingly, expressions of pathogenesis-related 4 (PR4) protein and plant defensin 1.3 (PDF1.3) were already induced at 4 hpi in the plant epidermis. PDF1.3 is an antifungal secretory protein [20], and PR-4 is an antifungal chitin-binding hevein-like protein [21]. Thus, the pathogen challenge rapidly activated the defense signaling and induced the expression of many defense-related proteins, including antifungal proteins, in epidermal tissues.

As shown in Figure 4, the number of mapped proteins increased from 4 to 24 hpi. At 24 hpi, many transcription factors, such as CPC, bZIP9, and CRF5, were up-regulated specifically in the leaf epidermis. CPC regulated the epidermal cell fate and promoted stomata formation in the hypocotyl [22,23]. The ethylene-responsive transcription factor CRF5 binds to the GCC-box in the promoter of *PR* genes. In addition, 12 cell-wall-related proteins were also up-regulated. They contained two cellulose biosynthesis proteins, one cell wall protein, three cell wall degradation proteins, and six-cell wall modification proteins. To arrest fungal colonization or penetration, plants likely expressed many proteins involved in plant cell wall integrity maintenance and fungal cell wall degradation in the epidermal tissues. Furthermore, the expression of the BAK1 (BR1-associated receptor kinase) protein was also up-regulated in the leaf epidermis at 24 hpi. It was reported that BAK1 protein phosphorylated brassinosteroid insensitive 1 (BRI1) and activated PAMP-triggered immunity (PTI) through its interaction with receptor proteins such as FLS2 and EFR [24].

At 24 hpi, we also found the up-regulation of disease-resistance proteins belonging to two classes of the NBS-LRR family. The first class of proteins is NBS-LRR, containing the TIR domain at the N-terminus (TIR-NBS-LRR), and the second class of proteins is CC-NBS-LRR [25]. These proteins contained Toll/interleukin-1 receptor (TIR)-nucleotide binding site–leucine-rich repeat (NBS-LRR) class disease resistance protein (AT4G09430.1), NBS-LRR with a coiled-coil domain (CC-NBS-LRR) class disease resistance protein (AT3G46730.1), and defender against apoptotic death 1 (DAD1; AT1G32210.1). It was reported that the NBS-LRR domain plays a crucial role in recognizing a broad range of pathogen effectors and subsequently induced effector-triggered immunity (ETI).

### 2.4. Proteome Data Were Confirmed Using Western Blotting

**Figure 5:**
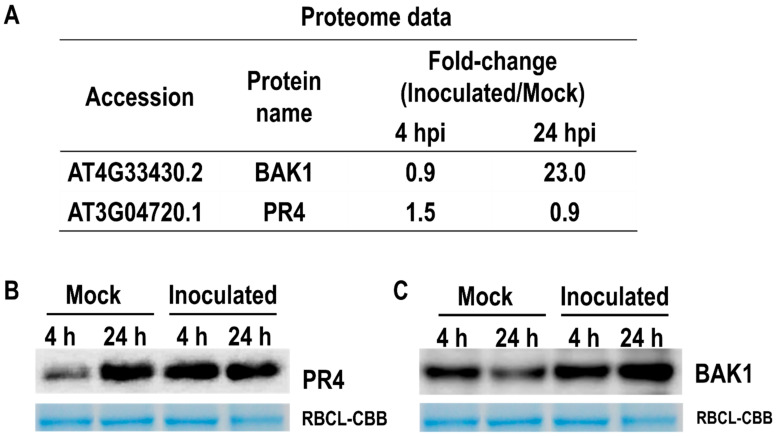
Protein accumulation was confirmed by immunoblotting analysis. (**A**) Proteome data of two accumulated proteins were based on LC-MS/MS analysis. (**B**,**C**) show that the accumulation of PR4 and BAK1 proteins can be confirmed by Western blotting analysis, respectively.

To validate the proteome data from LC-MS/MS analysis, we performed Western blotting of two up-regulated proteins of the leaf epidermis using the available antibodies against PR4 and BAK1. As shown in Figure 5A, PR4 protein was up-regulated by fungal inoculation at 4 hpi. On the other hand, BAK1 expression was increased at 24 hpi. We confirmed the expression of PR4 and BAK1 protein at 4 and 24 hpi. Figure 5B shows that the accumulation of PR4 protein was increased at 4 hpi. The expression of the BAK1 protein was clearly induced at 24 hpi.

### 2.5. Fungal Proteins Specifically Expressed on the Leaf Surface

Supplementary Figure S3 shows the protein preparation of fungal proteins expressed on the plant epidermis and fungal control proteins. As stated above, the same proteomic data from the epidermal peels with or without fungal inoculation were analyzed using the protein database of *Fusarium graminearum*. To identify fungal proteins specifically expressed on the leaf surface, we prepared protein extracts of fungal conidia before inoculation at 0 h as a control. As stated above, we identified many Arabidopsis proteins in the epidermal tissues of inoculated leaves. In contrast, the number of fungal proteins identified in the leaf epidermis was relatively low (Supplementary Figure S4). Protein extracts prepared from epidermal tissues contained many Arabidopsis proteins, including high-molecular-weight proteins. After trypsin digestion, large amounts of peptides derived from Arabidopsis proteins likely affected the detection of fungal proteins. Nonetheless, we tried to identify fungal proteins specifically expressed in the leaf epidermis and compared them with conidial proteins before inoculation (0 h). A total of 61 proteins were specifically expressed in the leaf epidermis at 4 and 24 hpi (Supplementary Figure S4). Because these proteins were not expressed in fungal conidia at 0 h, we believe these proteins were expressed upon contact with the leaf surface, followed by incubation for 4 or 24 h. Thus, these proteins might be important for fungal infection of the leaf epidermis. Among the 61 proteins, 55 were expressed at both time points (4 and 24 hpi), whereas 6 proteins were specifically expressed at 24 hpi.

Supplementary Table S3 summarizes all 61 fungal proteins identified in the epidermis. Two of these proteins (an SGNH-hydro domain protein and an uncharacterized protein) identified at 24 hpi contained signal peptides, indicating that they are secreted proteins. TauD domain- and NmrA domain-containing proteins were also specifically identified at 24 hpi; these proteins may be involved in fungal metabolism under stress conditions and in cell development, respectively. Supplementary Table S3 also lists 55 proteins commonly expressed at both time points and their ontologies based on the UniProt database. These included proteins involved in the response to oxidative stress, such as nitroreductase-domain- and thioredoxin-domain-containing proteins and thiamine thiazole synthase protein. We also identified glutathione C-terminal domain-containing proteins involved in glutathione metabolism. Two fork-head transcription factor proteins, required for mycelial growth and conidial germination of *Magnaporthe oryzae*, were also identified. These transcription factors are also crucial for fungal pathogenicity [26]. We identified fork-head-domain-containing proteins at both 4 and 24 hpi; these proteins may contribute to the conidial germination and mycelial growth of *F. graminearum* on Arabidopsis leaf epidermis. In addition, the SGNH-hydro domain-containing protein is commonly found in fungal rhamnogalacturonan acetylesterase and has been confirmed to be secreted by *F. graminearum* on the cell wall [27]. Thus, we identified fungal proteins specifically expressed upon contact with the leaf surface. These proteins potentially play important roles in fungal growth and pathogenicity.

## 3. Discussion

In this study, epidermal proteomics enabled the identification of many differentially expressed proteins related to plant–pathogen interactions. As mentioned above, pathogen attacks were first recognized by plant extracellular receptor proteins such RLKs or RLPs. The expression of the BAK1 protein was specifically up-regulated by fungal inoculation in the leaf epidermis at 24 hpi. Although BAK1 was often reported as a target of bacterial effectors such as flg22, Irieda reported that a fungal effector, necrosis-inducing secreted protein 1 (NIS1), interacts with BAK1 and then suppressed its kinase activity and immune response. Other RLKs such as PXY (TDR), cysteine-rich RLK proteins (CRK39), and CRK9 were up-regulated at 4 hpi [28]. CRK39 was annotated as a membrane-bound protein kinase in protein phosphorylation. CRK9 was thought to be an apoplast protein kinase involved in programmed cell death and systemic acquired resistance, depending on the Enhance Disease Susceptibility 1 (EDS1) protein [29]. In addition, the expression of the receptor for activated C kinase (RACK) 1B, which is involved in plant development, was also induced at 4 hpi. RACK1B was functionally redundant with RACK1C and RACK1A. The lethal phenotype was observed in the rack1a rack1b rack1c triple mutant [30]. It was also reported that RACK1 acted downstream of the G-protein and upstream of the MPK3/6; this pathway was activated by the pathogen-secreted protease of *Pseudomonas aeruginosa* [31]. The wall-associated receptor kinase carboxy-terminal protein (AT3G17350.1) was also induced at 4 hpi. Thus, expressions of protein kinases were induced in the inoculated epidermal tissues at 4 hpi, and most of them were likely involved in the plant immune response.

At 24 hpi, some RLK proteins were also up-regulated in the leaf epidermis, such as CRK2, GSO1, and proline-rich RLK (PERK15). Together with GSO2, the GSO1 protein participates in forming the epidermal surface at the embryo and seedling stages in Arabidopsis. The double mutant of these genes showed an abnormal stomatal pattern, size, and distribution [32]. The identified epidermal proteins also contained regulators of stomatal closure and opening, guard cell fate, and epidermal tissue differentiation. At 4 hpi, the IQ motif-containing protein 1 (IQM1), C-terminal binding protein AN (ANGUSTIFOLIA, CtBP), and integrin-linked protein kinase family were up-regulated (Supplementary Table S1). Proteins containing the IQ motif (IQxxxRGxxxR) are highly expressed in guard cells and respond to environmental cues. The IQM1-overexpressed transgenic plants showed a smaller stomatal aperture than that of the wild type [10]. In addition, the *iqm1* mutant accumulated reactive oxygen species (ROS) in guard cells. These reports suggested that the IQM1 gene was involved in regulating stomatal movement in response to biotic and abiotic stresses [10]. A knockout mutant of the AN gene showed distinct leaf morphology, decreased branching of trichomes on the leaf epidermis, and abiotic- and biotic-stress-resistant phenotypes compared with the wild type [33]. Myrosinase 2 (TTG2), which was involved in glucosinolate metabolism, was up-regulated specifically at 24 hpi in the leaf epidermis. The TGG2 gene was functionally redundant with TGG1 and regulated stomatal closure by methyl jasmonate and ABA treatments [34]. Thus, these proteins contained important regulators involved in regulating stomatal movement in response to pathogen attacks.

Generally, when a fungal pathogen successfully penetrates the host cells, it overcomes the plant defense by producing virulence factors such as effector-like proteins [35]. As shown in Supplementary Table S3, 5 effector-like proteins were predicted using EffectorP 2.0 [36] and expressed at both time points (4 and 24 hpi). The Rab7 protein was a key regulator of FgAtg9, and ATG9 is essential for the hyphal development and pathogenicity of *F. graminearum* [37]. In addition, the secreted effector-like SGNH-hydro domain-containing protein potentially plays an important role in fungal development and virulence [27]. GO enrichment analysis of 61 fungal proteins showing the enriched annotations related to accumulated proteins in the list compared to the database. Table 3 provides a list of the GO terms in various GO categories with a cutoff *p*-value < 0.05. The 19 enriched GO terms contained “nucleotide binding” and “kinase activity”. Four kinase domain-containing proteins were classified into GO terms such as “serine/threonine kinase active site” and “protein kinase domain.” Among these proteins, two, including I1RW1 (FGSG_08468) and a MAPK protein (I1RQN9; FGSG_06385), are essential for the vegetative growth and pathogenicity of *F. graminearum* on wheat spikes. A knockout mutant of the *I1RW1* gene showed a >30% lower mycelium growth rate on the complete medium. Additionally, the disease index was decreased by 80% on the wheat spike inoculated with this mutant at 14 days post-inoculation compared with the wild type. In addition, the *I1RQN9* knockout mutation reduced the mycelium growth and virulence in wheat spikes compared with the wild type [38]. Thus, our identified fungal proteins specifically expressed on the leaf epidermis likely contained the important regulators in the plant and pathogen interactions.

Thaumatin-like proteins are another group of plant proteins that play crucial roles in FHB resistance in wheat and barley [39,40]. These proteins are induced by *F. graminearum* infection and accumulate in FHB-resistant genotypes. In this study, we also identified six thaumatin superfamily proteins at 4 hpi and 24 hpi. However, the abundance of these proteins was not increased by the inoculation of *F. graminearum*. Since the leaf of Arabidopsis (Col-0) was susceptible to *F. graminearum*, up-regulation of thaumatin-like proteins might be observed preferentially in *F. graminarum*-resistant plants [41].

In this study, we revealed that the epidermal proteomic approach is useful for identifying differentially expressed proteins on the plant surface after a pathogen challenge. The expression of most of their proteins was not significantly changed in the whole leaves. If epidermal tissues can be prepared, epidermal proteomics broadly applies to various plant materials. Rapid and local protein expression during the plant–pathogen interaction can be easily monitored by epidermal proteomics. As stated above, our identified proteins potentially contained many important regulators both in plants and pathogens. Moreover, since epidermal proteomics is a simple method, the differential expression pattern of plant and fungal proteins can be compared among different types of plant and/or pathogens (i.e., host vs. non-host resistance, hemibiotrophic vs. necrotrophic pathogens). In addition, epidermal proteomics applies to many plant species and tissues if epidermal tissues can be prepared. Interestingly, some published plant epidermal proteomics studies showed that many defense-related proteins are already expressed without pathogen challenge. This implies that epidermal plant tissues are vital as the first line of defense against pathogens and herbivores. Furthermore, dynamic protein communication between plants and pathogens can be accurately measured by epidermal proteomics. Future genetic studies on both plants and pathogens will also support the utility of epidermal proteomics.

## 4. Materials and Methods

### 4.1. Plant Materials and Growth Conditions

Seeds of *A. thaliana* ecotype Columbia (Col-0) were sown on soil and vernalized for 2 days in the dark at 4 °C. The seedlings were grown in soil under long-day photoperiod (16 h light/8 h dark) for 4 to 5 weeks. Mature leaves of 4- to 5-week-old plants were used for the fungal-inoculation assay [42].

### 4.2. Fungal Conidia Preparations and Inoculations

*Fusarium graminearum* strain H3 was cultured in liquid synthetic nutrient medium (0.1% (*w*/*v*) KH_2_PO_4_, 0.1% (*w*/*v*) KNO_3_, 0.1% (*w*/*v*) MgSO_4_∙7H_2_O, 0.05% (*w*/*v*) KCl, 0.02% (*w*/*v*) glucose, 0.02% (*w*/*v*) sucrose) at 22 °C for 3 days with constant shaking, as previously described [43]. Then, the culture solution was filtered through a cell strainer with a 100 µm pore size. After filtration, the fungal cultures were centrifuged at 15,000 rpm for 5 min, and pellets were washed with 1× phosphate-buffered saline (PBS). The washing steps were repeated three times, and the pellets were suspended by PBS buffer. The number of collected conidia was counted under the microscope (Olympus BX-50) using a hemocytometer.

Fungal-inoculation assay was performed by drop inoculation to the detached leaves, as previously described [42], with slight modifications. Briefly, leaves of 4- to 5-week-old Arabidopsis plants were cut and then arranged in a square dish with moistened Kimberly-Clark towels to keep high humidity. Five µL of the conidial suspension (1 × 10^5^ conidia/mL) containing 0.001% (*v*/*v*) Silwet L-77 was dropped onto the leaves and covered with a piece of nylon mesh (3 mm × 3 mm square). Mock-treated leaves were inoculated with 5 µL of PBS without conidia.

### 4.3. Visualization and Quantification of the Fungal Pathogen on the Leaf Surface

The inoculated leaves were fixed using the fixation solution (75% acetic acid and 25% ethanol), subsequently degassed for 20 min, and washed three times using PBS. Then, the fungal mycelia were stained with wheat germ agglutinin (WGA)-Alexa fluor 488 conjugate (ThermoFisher Scientific, Waltham, MA, USA) and observed under a fluorescence microscope (AZ100M; Nikon, Tokyo, Japan) and a confocal laser scanning microscope (LSM5 PASCAL; Carl Zeiss, Jena, Germany). Genomic DNA (gDNA) was isolated from the epidermis of the inoculated leaves using the Nucleon Phytopure Genomic DNA Extraction Kit (GE Healthcare, Tokyo, Japan). The concentration of the isolated gDNA was adjusted to 100 ng/µL and used as the DNA template for quantitative real-time PCR. The amounts of fungal gDNA were estimated as the ratio of the fungal EF-1α gene to the total gDNA, including Arabidopsis Act2/8 and fungal EF-1α gene). The primers used to amplify the fungal genes were EF1α_F-CCATTCCCTGGGCGCT and EF1α_R-CCTATTGACAGGTGGTTAGTGACTGG, whereas those used to amplify the plant gene were Act2/8_F-GGTAACATTGTGCTCAGTGGTGG and Act2/8_R-AACGACCTTAATCTTCATGCTGA [44].

### 4.4. Protein Preparation from Leaf Epidermis

The experimental procedures of sample preparation are shown in Figure 2A. The leaf epidermis was collected from inoculated leaves at 0, 4, and 24 hpi. Nylon mesh was removed from the leaf surface, and the leaves were placed on double-sided tape. The leaf epidermis was peeled using sharp tweezers within 3 min. The collected epidermis was placed in 2 mL sample tubes floating on liquid nitrogen and crushed into fine powders using the beads crusher (Shake Master Neo, BMS-M10N21, Biomedical Science Co., Ltd., Tokyo, Japan). Each sample contained epidermal peels from ten leaves, and four replicates were prepared for each treatment.

To extract proteins from the collected epidermis, 100 µL of the protein extraction buffer (1× PBS, 1% Triton-X100, and 1× proteinase inhibitor cocktail) was added to the fine powders of leaf epidermis. They were centrifuged at 15,000 rpm for 10 min at 4 °C, and the supernatants were purified to remove Triton-X100 using a detergent-removal column (Thermo Scientific, San Jose, CA, USA). Protein concentrations were measured using the BCA protein assay kit (Takara Bio, Shiga, Japan).

### 4.5. Shotgun Proteomics

A total of 50 ug of protein samples was dried using the savant speed-vac. The resulting pellets were resuspended in 6M Urea and 50 mM TEAB (triethylammonium bicarbonate) at a pH of 8.5. Proteins samples were adjusted to a final volume of 10 µL, reduced with 5 mM TCEP for 30 min at 37 °C in the dark, and alkylated with 24 mM iodoacetamide for 30 min at r.t. in the dark. Alkylated proteins were digested with trypsin (Mass spectrometry grade, Promega, Japan) at a 1:10 enzyme/protein ratio for 16 h at 37 °C. Peptides were desalted with Stage tip #84850 (Thermo Pierce, Tokyo, Japan) and eluted with 70% ACN. Then, eluted peptides were dried by vacuum centrifuge and dissolved with 5% ACN containing 0.1% trifluoroacetic acid.

The trypsin-digested peptides were analyzed by Orbitrap QE plus (Thermo Fisher Scientific) with nano-liquid chromatography (EASY-nLC 1200; Thermo Fisher Scientific). The purified peptides were loaded and separated on the column (25 cm × 75 µm ID, 1.6 mm C18; Ionoptics) with a linear acetonitrile gradient (0–35%) in 0.1% formic acid at a flow rate of 300 nL min^−1^. The peptide ions were detected by Orbitrap QE plus MS; Thermo Fisher Scientific) in the data-dependent acquisition mode with the installed Xcalibur software (Thermo Fisher Scientific). Full-scan mass spectra were acquired in the MS over 375-1500 *m*/*z* with a resolution of 70,000.

The MS/MS searches were conducted using SEQUEST HT search algorithms against the TAIR Arabidopsis protein database using Proteome Discoverer (PD) 2.2 (Version 2.2.0.388; Thermo Fisher Scientific). Label-free quantification was also performed with PD 2.2 using precursor ions quantifier nodes. The processing workflow included spectrum files RC, spectrum selector, SEQUEST HT search nodes, percolator, ptmRS, and minor feature-detector nodes. Methionine oxidation was set as a variable modification, and carbamidomethylation of cysteine was set as a fixed modification. Mass tolerances in MS and MS/MS were set at 10 ppm and 0.6 Da, respectively. Trypsin was specified as protease, and a maximum of two missed cleavages were allowed. Target-decoy database searches were used to calculate the false-discovery rate (FDR), and peptide identification FDR was set at 1%.

Label-free quantification was also performed with PD 2.2 using precursor ions quantifier nodes. The consensus workflow included MSF files, Feature Mapper, precursor ion quantifier, PSM groper, peptide validator, peptide and protein filter, protein scorer, protein marker, protein FDR validator, protein grouping, and peptide in protein. Normalization of the abundances was performed using the total peptide amount mode. Gene Ontology (GO) enrichment analysis was performed according to DAVID Bioinformatics Resources 6.8 [45]. The fungal effector-like proteins were predicted by EffectorP 2.0 (http://effectorp.csiro.au, accessed on 29 January 2021) [36]. Functional mapping analysis was conducted using MapMan bin codes of the TAIR 10 August 2012 database [14].

### 4.6. Immunoblotting Analysis

Ten micrograms of proteins were denatured with an SDS sample buffer at 95 °C for 5 min. Then, proteins were separated by electrophoresis on a 5–12% SDS polyacrylamide gel with a precision plus protein standard (Bio-Rad, Hercules, CA, USA). The proteins in polyacrylamide gels were transferred to PVDF membranes using the transblot SD cell (Biorad, Tokyo, Japan). Blotted membranes were blocked using 5% skim milk for 1 h with shaking. The anti-BAK1 (Agrisera, AS12 1858) and anti-PR4 (Agrisera, AS12 2369) antibodies were used as primary antibodies. Antirabbit IgG conjugated with horseradish peroxidase (Cytiva Global Life Science Technologies Japan Co., Ltd., Japan) was used as the secondary antibody. These protein signals were detected by ECL Select detection kit (GE Healthcare, Japan) using LAS500 chemiluminescence equipment (GE Healthcare, Japan).

## Figures and Tables

**Table 1:** GO enrichment analysis of highly up-regulated Arabidopsis proteins specifically in the epidermis at 4 hpi.

Enrichment FDR	Number of Proteins	Functional Category
3.4 × 10^−2^	16	Translation
3.4 × 10^−2^	16	Peptide biosynthetic process
3.4 × 10^−2^	17	Amide biosynthetic process
4.0 × 10^−2^	7	Defense response, incompatible interaction
4.4 × 10^−2^	16	Peptide metabolic process

**Table 2:** GO enrichment analysis of up-regulated proteins specifically in the leaf epidermis at 24 hpi.

Enrichment FDR	Number of Proteins	Functional Category
8.4 × 10^−7^	30	Amide biosynthetic process
8.4 × 10^−7^	46	Organonitrogen compound biosynthetic process
1.0 × 10^−6^	28	Translation
1.0 × 10^−6^	28	Peptide biosynthetic process
5.0 × 10^−6^	28	Peptide metabolic process
8.2 × 10^−6^	30	Cellular amide metabolic process
6.6 × 10^−4^	32	Cellular component biogenesis
2.8 × 10^−3^	23	Cellular component assembly
3.4 × 10^−3^	19	Protein-containing complex assembly
5.3 × 10^−3^	17	Cellular-protein-containing complex assembly
1.1 × 10^−2^	55	Cellular component organization or biogenesis
1.2 × 10^−2^	19	Protein-containing complex subunit organization
2.0 × 10^−2^	8	Organelle assembly
2.9 × 10^−2^	12	Response to cold
2.9 × 10^−2^	49	Cellular component organization
3.2 × 10^−2^	5	Cytoplasmic translation
3.2 × 10^−2^	6	Translational initiation
3.2 × 10^−2^	15	Response to temperature stimulus
3.2 × 10^−2^	35	Response to abiotic stimulus
3.6 × 10^−2^	63	Protein metabolic process
3.7 × 10^−2^	58	Cellular protein metabolic process
5.0 × 10^−2^	12	Ribonucleoprotein complex biogenesis

**Table 3:** GO enrichment analysis of 61 fungal proteins, including 6 proteins specifically identified at 24 hpi and 55 proteins commonly identified at 4 and 24 hpi. UP: UniProt, BP: biological process, CC: cellular component, MF: molecular function, KEGG: Kyoto Encyclopedia of Genes and Genomes. Cutoff *p*-value < 0.05.

Category	Terms	*p*-Value
UP_KEYWORDS	ATP-binding	7.3 × 10^−6^
UP_KEYWORDS	Nucleotide-binding	2.6 × 10^−5^
UP_KEYWORDS	Kinase	4.1 × 10^−3^
UP_KEYWORDS	Transferase	4.3 × 10^−3^
UP_KEYWORDS	Serine/threonine-protein kinase	6.5 × 10^−3^
GOTERM_CC	Cytosol	6.7 × 10^−3^
INTERPRO	Serine/threonine-protein kinase, active site	6.9 × 10^−3^
GOTERM_MF	ATP binding	7.6 × 10^−3^
INTERPRO	Acetate-CoA ligase	8.9 × 10^−3^
GOTERM_BP	Acetyl-CoA biosynthetic process from acetate	1.1 × 10^−2^
GOTERM_MF	Acetate-CoA ligase activity	1.2 × 10^−2^
GOTERM_MF	AMP binding	1.2 × 10^−2^
SMART	S_TKc	1.4 × 10^−2^
UP_KEYWORDS	Ligase	1.5 × 10^−2^
INTERPRO	Protein kinase, catalytic domain	1.8 × 10^−2^
GOTERM_BP	Negative regulation of sequence-specific DNA binding transcription factor activity	2.1 × 10^−2^
INTERPRO	Protein kinase, ATP binding site	2.9 × 10^−2^
KEGG_PATHWAY	Biosynthesis of antibiotics	3.0 × 10^−2^
INTERPRO	Protein kinase-like domain	4.4 × 10^−2^

## Data Availability

The data presented in this study are available online within this article or in the Supplementary Materials.

## Supplementary Materials

The following supporting information can be downloaded at: https://www.mdpi.com/article/10.3390/ijms232012171/s1.
