# Supplementary material for: Proteomic Profiling of Plant and Pathogen Interaction on the Leaf Epidermis
Source: Int J Mol Sci. 2022 Oct 12;23(20):12171. doi: 10.3390/ijms232012171 (PMC9603099; doi:10.3390/ijms232012171)
Supplement: Supplementary file 1 [file ijms-23-12171-s001.zip › ijms-1947777-supplementary/Supplementary/Supplementary FIgures_EP.pptx]

## Slide 1
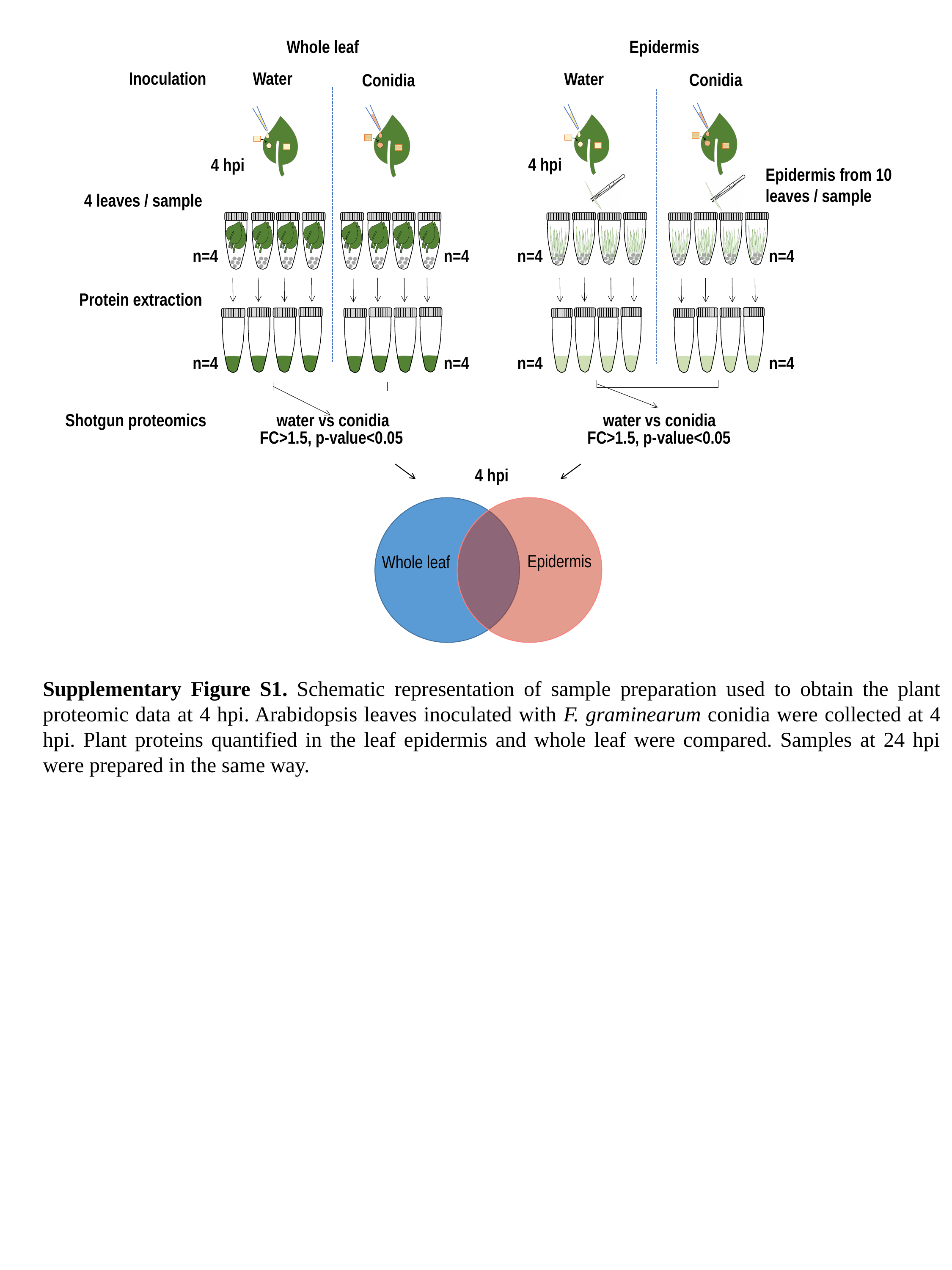

Whole leaf
Epidermis
Inoculation
Water
Water
Conidia
Conidia
4 hpi
4 hpi
Epidermis from 10 leaves / sample
4 leaves / sample
n=4
n=4
n=4
n=4
Protein extraction
n=4
n=4
n=4
n=4
Shotgun proteomics
water vs conidia
water vs conidia
FC>1.5, p-value<0.05
FC>1.5, p-value<0.05
4 hpi
Epidermis
Whole leaf
Supplementary Figure S1. Schematic representation of sample preparation used to obtain the plant proteomic data at 4 hpi. Arabidopsis leaves inoculated with F. graminearum conidia were collected at 4 hpi. Plant proteins quantified in the leaf epidermis and whole leaf were compared. Samples at 24 hpi were prepared in the same way.

## Slide 2
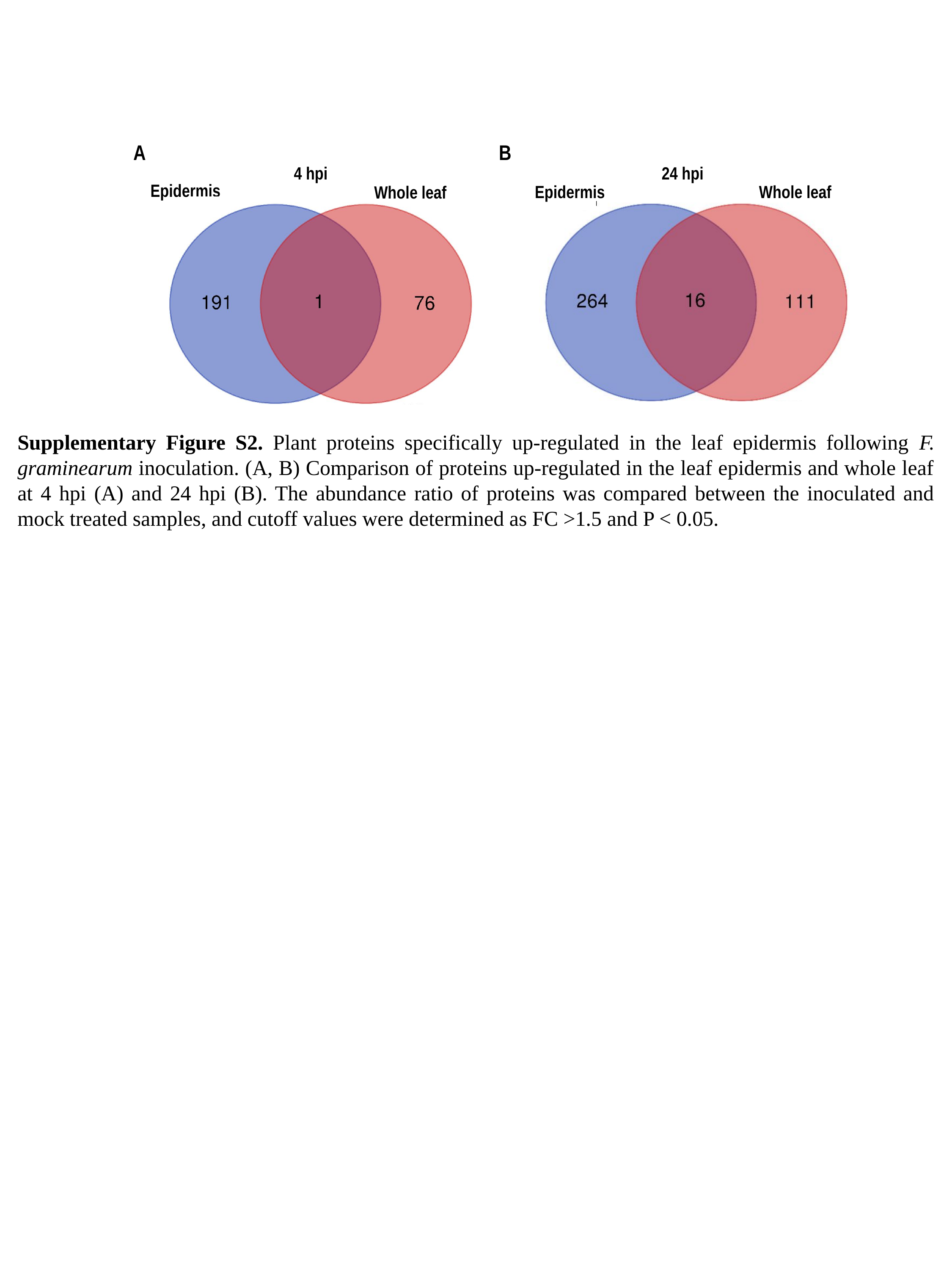

A
B
4 hpi
24 hpi
Epidermis
Whole leaf
Epidermis
Whole leaf
Supplementary Figure S2. Plant proteins specifically up-regulated in the leaf epidermis following F. graminearum inoculation. (A, B) Comparison of proteins up-regulated in the leaf epidermis and whole leaf at 4 hpi (A) and 24 hpi (B). The abundance ratio of proteins was compared between the inoculated and mock treated samples, and cutoff values were determined as FC >1.5 and P < 0.05.

## Slide 3
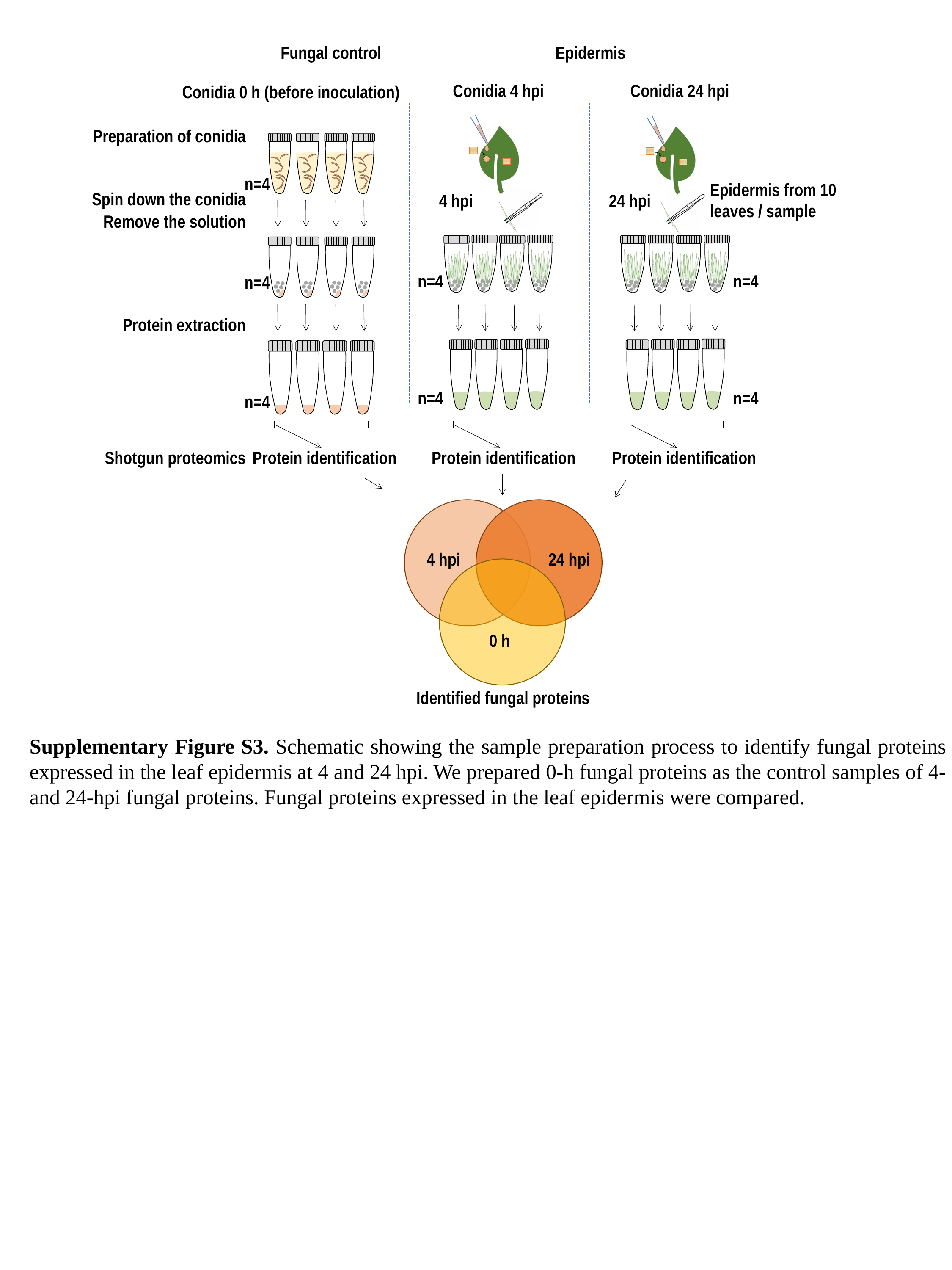

Fungal control
Epidermis
Conidia 4 hpi
Conidia 24 hpi
Conidia 0 h (before inoculation)
Preparation of conidia
n=4
Epidermis from 10 leaves / sample
Spin down the conidia
4 hpi
24 hpi
Remove the solution
n=4
n=4
n=4
Protein extraction
n=4
n=4
n=4
Shotgun proteomics
Protein identification
Protein identification
Protein identification
24 hpi
4 hpi
0 h
Identified fungal proteins
Supplementary Figure S3. Schematic showing the sample preparation process to identify fungal proteins expressed in the leaf epidermis at 4 and 24 hpi. We prepared 0-h fungal proteins as the control samples of 4- and 24-hpi fungal proteins. Fungal proteins expressed in the leaf epidermis were compared.

## Slide 4
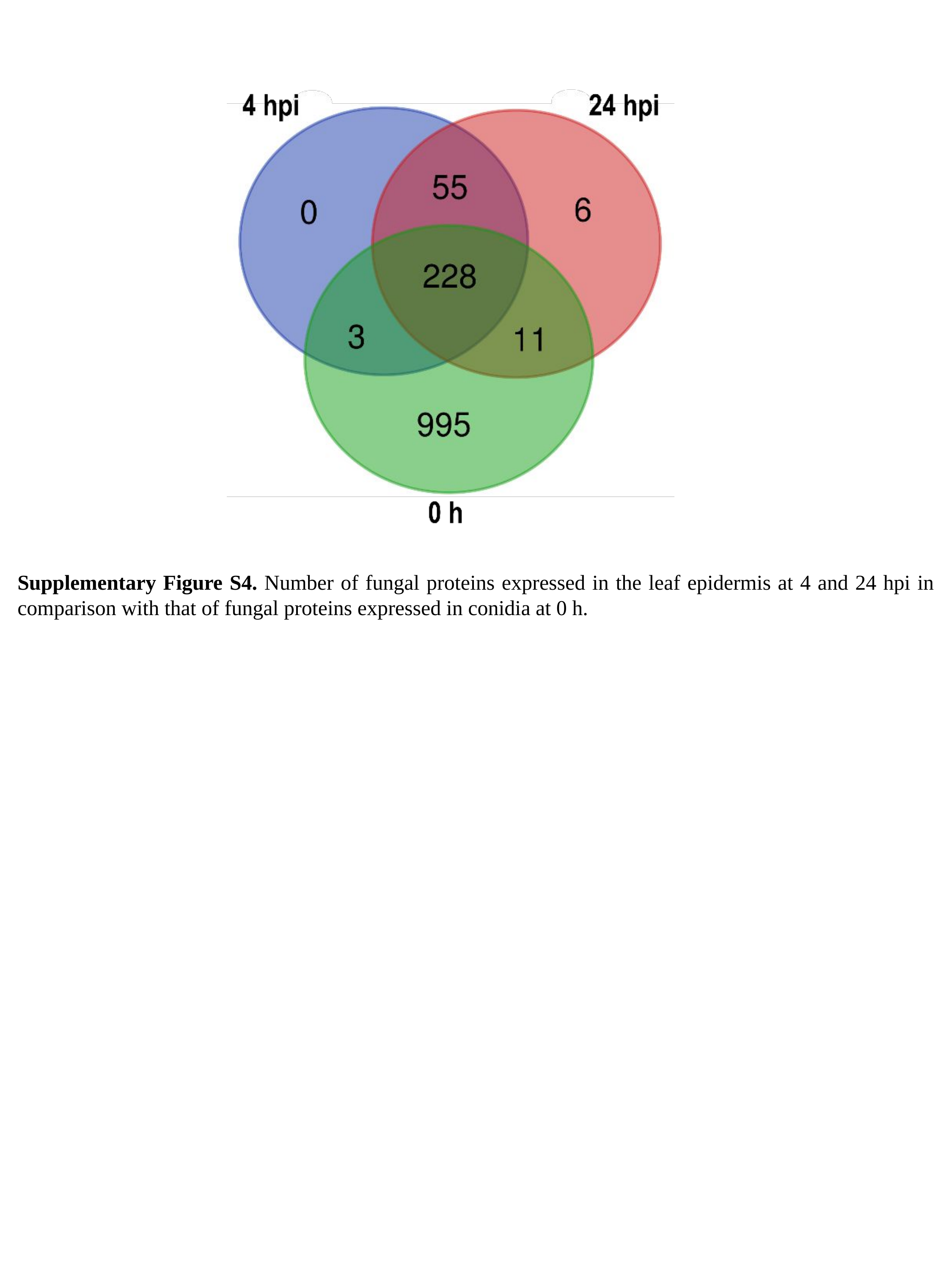

Supplementary Figure S4. Number of fungal proteins expressed in the leaf epidermis at 4 and 24 hpi in comparison with that of fungal proteins expressed in conidia at 0 h.
