# Supplementary material for: Proteomic Profiling of Plant and Pathogen Interaction on the Leaf Epidermis
Source: Int J Mol Sci. 2022 Oct 12;23(20):12171. doi: 10.3390/ijms232012171 (PMC9603099; doi:10.3390/ijms232012171)
Supplement: Supplementary file 1 [file ijms-23-12171-s001.zip › ijms-1947777-supplementary/Supplementary/Supplementary tables_EP.pdf]

Supplementary Table S1. List of proteins up-regulated in the leaf epidermis at 4 hpi. Asterisk (*) indicates proteins up-regulated in the leaf epidermis as well as in the whole leaf.

Accession	Protein names	Fold-change	p-value
**3 Proteins related to stomatal closure and epidermal cell fate**			
AT4G33050.2	IQ domain-containing protein IQM1	100	4.21.E-16
AT1G01510.1	C-terminal binding protein AN (CtBP)	3.6	2.32.E-06
AT4G18950.1	Integrin-linked protein kinase family	1.9	2.81.E-02
**7 Receptor proteins**			
AT4G04540.1	Putative cysteine-rich receptor-like protein kinase 39	100	4.21.E-16
AT2G01070.1	Lung seven transmembrane receptor family protein	12.3	4.21.E-16
AT1G68400.1	Probable leucine-rich repeat receptor-like protein kinase	3.1	2.26.E-02
AT3G17350.1	Wall-associated receptor kinase carboxy-terminal protein	2.3	1.31.E-02
AT4G23170.1	Putative cysteine-rich receptor-like protein kinase 9	1.6	7.57.E-04
AT5G61480.1	Leucine-rich repeat receptor-like protein kinase TDR	7.4	9.27.E-15
AT1G48630.1	Receptor for activated C kinase 1B	1.6	1.72.E-03
**4 Plant type cell wall and cell wall organization**			
AT5G22740.1	Glucomannan 4-beta-mannosyltransferase 2	2.8	2.79.E-07
AT4G19420.1	Pectin acetylesterase 8	2.1	8.04.E-03
AT5G18470.1	Curculin-like (Mannose-binding) lectin family protein	1.6	4.00.E-02
AT2G20810.1	Probable galacturonosyltransferase 10	1.9	2.02.E-02
**31 Transmembrane proteins (transport-related)**			
AT1G14140.1	Mitochondrial uncoupling protein 3 (AtPUMP3)	100	4.21.E-16
AT1G16020.1	Vacuolar fusion protein CCZ1 homolog A	100	4.21.E-16
AT1G72820.1	At1g72820/F3N23_2 (F3N23.2 protein)	100	4.21.E-16
AT2G32720.1	Cytochrome b5 isoform B (AtCb5-B)	100	4.21.E-16
AT2G39130.1	Amino acid transporter AVT1C (AtAvt1C)	100	4.21.E-16
AT5G40930.1	Mitochondrial import receptor subunit TOM20-4	100	4.21.E-16
AT1G17200.1	CASP-like protein 2A1 (AtCASPL2A1)	100	4.21.E-16
AT4G39890.1	Ras-related protein RABH1c (AtRABH1c)	9.6	4.21.E-16
AT3G11170.1	sn-2 acyl-lipid omega-3 desaturase (ferredoxin)	7.8	2.08.E-12
AT1G27695.1	Protein TRIGALACTOSYLDIACYLGLYCEROL 5	5.3	2.70.E-07
AT5G42960.1	Outer envelope pore protein 24B	5.0	9.03.E-13
AT4G00430.1	Probable aquaporin PIP1-4	4.3	8.71.E-13
AT1G69680.1	At1g69680/T6C23_12	3.4	8.14.E-10
AT3G08710.1	Thioredoxin H9 (AtTrxh9)	3.4	7.01.E-07
AT5G55710.1	Protein TIC 20-v	3.3	1.98.E-04
AT2G31710.1	Vacuolar ATPase assembly integral membrane	3.2	4.27.E-06

Accession	Protein names	Fold-change	p-value
AT1G04980.1	Protein disulfide-isomerase like 2-2	2.9	7.84.E-04
AT2G39210.1	At2g39210/T16B24.15 (Nodulin-like protein)	2.9	2.44.E-03
AT1G08380.1	Photosystem I subunit O (PSI-O)	2.6	8.80.E-07
AT5G45750.1	Ras-related protein RABA1c (AtRABA1c)	2.6	6.91.E-03
AT1G20840.1	Monosaccharide-sensing protein 1	2.6	2.34.E-04
AT1G06210.1	TOM1-like protein 2	2.5	4.40.E-02
ATCG01070.1	NAD(P)H-quinone oxidoreductase subunit 4L	2.4	1.86.E-03
AT3G26570.1	Inorganic phosphate transporter 2-1	2.2	8.73.E-04
AT4G25760.1	Protein GLUTAMINE DUMPER 2	1.9	1.37.E-03
AT2G38410.1	TOM1-like protein 6	1.8	9.92.E-03
AT2G12400.1	Plasma membrane fusion protein	1.8	1.33.E-03
AT5G47200.1	Ras-related protein RABD2b (AtRABD2b)	1.7	3.58.E-04
AT2G36410.2	RAB6-interacting golgin	2.0	2.46.E-03
AT3G01050.1	Membrane-anchored ubiquitin-fold protein 1 (AtMUB1)	3.1	2.19.E-08
ATCG00580.1	Cytochrome b559 subunit alpha	1.6	3.83.E-03
**12 Defense response proteins**			
AT1G77470.1	Replication factor C subunit 5 (AtRFC5)	100	4.21.E-16
AT2G30740.1	PTI1-like tyrosine-protein kinase 2 (PTI1-2)	100	4.21.E-16
AT3G21220.1	Mitogen-activated protein kinase kinase 5	100	4.21.E-16
AT5G54380.1	Receptor-like protein kinase THESEUS 1	100	4.21.E-16
AT5G38900.1	FrnE protein-like (Putative frnE protein)	2.5	3.22.E-03
AT3G15020.1	Malate dehydrogenase 2	2.4	3.09.E-10
AT3G19380.1	U-box domain-containing protein 25	2.3	3.17.E-10
AT1G17420.1	Lipoxygenase 3, chloroplastic (AtLOX3)	2.0	1.10.E-02
AT4G09430.1	Disease resistance protein (TIR-NBS-LRR class)	5.9	4.21.E-16
AT3G04720.1	Hevein-like preproprotein	1.5	4.14.E-04
AT2G26010.1	Plant defensin 1.3	7.2	4.21.E-16
AT4G16260.1	Probable glucan endo-1,3-beta-glucosidase At4g16260	1.5	4.22.E-03
**9 Proteins related to abiotic stress response and detoxification**			
AT4G16440.1	Protein NAR1 (Nuclear architecture related 1)	100	4.21.E-16
AT2G04080.1	Protein DETOXIFICATION 2 (AtDTX2)	5.2	5.62.E-06
AT2G47180.1	Galactinol synthase 1 (AtGolS1)	3.1	4.83.E-06
AT4G04885.1	Polyadenylation and cleavage factor homolog 4	2.4	8.71.E-04
AT1G54410.1	Dehydrin HIRD11 (AtHIRD11)	2.0	8.24.E-06
AT3G03640.1	Probable inactive beta-glucosidase 25 (AtBGLU25)	2.0	2.04.E-02
AT3G13100.1	Multidrug resistance-associated protein 7	100	4.21.E-16

Accession	Protein names	Fold-change	p-value
AT1G01620.1	Aquaporin PIP1-3 (AtPIP1;3)	6.1	4.21.E-16
AT2G22360.1	Chaperone protein dnaJ A6, chloroplastic (atDjA6)	1.6	1.36.E-02
**<u>7 Transcription factors and transcription related-proteins</u>**			
AT3G42790.1	PHD finger protein ALFIN-LIKE 3 (Protein AL3)	100	4.21.E-16
AT5G18960.1	Protein FAR1-RELATED SEQUENCE 12	100	4.21.E-16
AT2G04630.1	DNA-directed RNA polymerases II and V subunit 6B	9.1	1.70.E-08
AT4G13670.1	Protein disulfide isomerase pTAC5,	5.9	4.21.E-16
AT2G35110.1	Protein NAP1 (ARP2/3 regulatory protein subunit NAPP)	2.7	2.98.E-02
AT4G40030.2	Histone H3.3 (Histone H3.2)	1.5	3.76.E-03
AT2G33510.2	ATP-dependent RNA helicase (At2g33510)	2.4	4.06.E-03
**<u>14 Ribosomal proteins</u>**			
AT3G14600.1	60S ribosomal protein L18a-3	4.6	1.01.E-10
AT5G64670.1	Ribosomal protein L18e/L15 superfamily protein	4.0	1.79.E-05
AT5G56670.1	40S ribosomal protein S30	3.0	2.74.E-08
AT5G64140.1	40S ribosomal protein S28-2	2.9	4.41.E-06
AT1G07070.1	60S ribosomal protein L35a-1	2.5	1.37.E-08
AT3G43980.1	40S ribosomal protein S29	10	4.21.E-16
AT5G47930.1	40S ribosomal protein S27-3	7.3	4.21.E-16
AT3G53740.2	60S ribosomal protein L36-2	3.9	1.37.E-06
ATCG00065.1	30S ribosomal protein S12	1.7	9.00.E-04
AT5G57290.1	60S acidic ribosomal protein P3-2	1.9	3.32.E-02
AT5G02610.2	60S ribosomal protein L35-4	1.6	5.05.E-03
AT5G54600.1	50S ribosomal protein L24	1.6	2.30.E-02
AT1G09590.1	60S ribosomal protein L21-1	1.5	8.80.E-04
AT3G45030.1	40S ribosomal protein S20-1	1.5	1.01.E-03
**<u>3 Translation related proteins</u>**			
AT5G18110.1	Eukaryotic translation initiation factor NCBP	5.0	5.42.E-06
AT2G25800.1	Elongation factor Ts (DUF810)	2.6	2.93.E-04
AT4G10760.1	N6-adenosine-methyltransferase MT-A70-like	3.2	9.36.E-09
**<u>5 Proteinases</u>**			
AT3G26340.1	Proteasome subunit beta type-5-B	100	4.21.E-16
AT5G01800.1	AT5g01800/T20L15_70	100	4.21.E-16
AT5G51740.1	Peptidase family M48 family protein	100	4.21.E-16
AT4G16800.1	Probable enoyl-CoA hydratase 2	3.2	1.55.E-13
AT1G51760.1	IAA-amino acid hydrolase ILR1-like 4	1.7	1.33.E-05

Accession	Protein names	Fold-change	p-value
**<u>3 Enzymes inhibitors</u>**			
AT4G25260.1	Pectinesterase inhibitor 7 (AtPMEI7)	100	4.21.E-16
AT5G46950.1	At5g46950 (Plant invertase)	100	4.21.E-16
AT1G73260.1	Kunitz trypsin inhibitor 4 (AtKTI4)	1.5	1.46.E-02
**<u>8 Proteins related to plant growth and development</u>**			
AT5G64080.1	Non-specific lipid transfer protein GPI-anchored 31 (AtLTPG-31)	100	4.21E-16
AT5G56660.1	IAA-amino acid hydrolase ILR1-like 2	100	4.21E-16
AT3G09920.3	Phosphatidylinositol 4-phosphate 5-kinase 9 (AtPIP5K9)	100	4.21E-16
AT1G75750.1	Gibberellin-regulated protein 1 (GAST1 protein homolog 1)	3.0	2.32E-04
AT5G28750.1	Protein THYLAKOID ASSEMBLY 4	1.6	2.02E-02
AT3G23980.1	Protein BLISTER (Protein KOLD SENSITIV-1)	2.4	6.43E-03
AT2G46720.1	3-ketoacyl-CoA synthase 13 (KCS-13)	1.5	3.85E-02
AT1G05850.1	Chitinase-like protein 1 (AtCTL1)	1.9	9.43E-03
**<u>86 Protein with other</u>**			
AT1G03650.1	Acyl-CoA N-acyltransferases (NAT) superfamily protein	100	4.21E-16
AT1G22800.1	Putative methyltransferase At1g22800	100	4.21E-16
AT2G14835.1	Expressed protein (RING/U-box superfamily protein)	100	4.21E-16
AT2G15240.1	At2g15240/F15A23.2 (Expressed protein)	100	4.21E-16
AT2G30170.1	Probable protein phosphatase 2C 26 (AtPP2C26)	100	4.21E-16
AT3G03560.1	Uncharacterized protein	100	4.21E-16
AT3G12650.1	AT3g12650/T2E22_103 (Transmembrane protein)	100	4.21E-16
AT3G18270.1	Dipeptide epimerase (EC 5.1.1.-)	100	4.21E-16
AT3G22740.1	Homocysteine S-methyltransferase 3	100	4.21E-16
AT4G19100.1	Protein PAM68,	100	4.21E-16
AT4G19830.1	Peptidyl-prolyl cis-trans isomerase FKBP17-1	100	4.21E-16
AT4G31560.1	AT4g31560 (AT4g31560/F3L17_130)	100	4.21E-16
AT4G37010.2	Calcium-binding protein CML19 (AtCEN2)	100	4.21E-16
AT4G37310.1	Cytochrome P450, family 81	100	4.21E-16
AT5G23490.1	Uncharacterized protein	100	4.21E-16
AT5G36950.1	Protease Do-like 10, mitochondrial (EC 3.4.21.-)	100	4.21E-16
AT5G40340.1	Tudor/PWWP/MBT superfamily protein	100	4.21E-16
AT5G46580.1	Pentatricopeptide repeat-containing protein At5g46580	100	4.21E-16
AT5G47810.1	Phosphohexokinase 2	100	4.21E-16
AT4G31080.2	Integral membrane metal-binding family protein (DUF2296)	27.3	4.21E-16
AT5G63550.2	DEK domain-containing chromatin associated protein	18.8	4.21E-16
AT1G01725.1	Adenylosuccinate synthetase (T1N6.13 protein)	12.9	4.21E-16

Accession	Protein names	Fold-change	p-value
AT1G31150.1	F28K20.8 protein (K-box region protein (DUF1985))	12.8	4.21E-16
AT1G66820.1	Glycine-rich protein	10.3	4.21E-16
AT2G32980.1	AUGMIN subunit 2	9.5	4.21E-16
AT1G47720.1	Protein OSB1,	9.2	4.21E-16
AT4G00231.1	ARM repeat superfamily protein (F6N15.2 protein)	9.1	6.91E-13
AT1G14060.1	F7A19.14 protein (GCK domain-containing protein)	8.9	1.93E-09
AT1G47570.1	At1g47570 (RING/U-box superfamily protein)	8.7	3.05E-10
AT5G63620.1	AT5g63620/MBK5_9	7.0	1.04E-09
AT3G17365.2	S-adenosyl-L-methionine-dependent methyltransferases	6.5	4.21E-16
AT5G06220.1	LETM1-like protein	6.4	4.08E-06
AT5G64410.1	Oligopeptide transporter 4 (AtOPT4)	6.2	1.18E-09
AT4G34940.1	Armadillo repeat only 1	5.9	5.56E-07
AT3G10405.1	At3g10405 (Vacuolar acid trehalase)	5.9	4.21E-16
AT2G38050.1	Steroid 5-alpha-reductase DET2 (AtDET2)	5.5	4.21E-16
AT3G46630.1	DCL protein (DUF3223)	4.8	4.52E-11
AT5G52100.1	Dihydrodipicolinate reductase-like protein CRR1,	4.5	9.27E-15
AT4G31440.1	Transcriptional regulator of RNA polII, SAGA, subunit	4.4	4.21E-16
AT5G12240.1	At5g12240 (Octanoyltransferase)	4.2	4.21E-16
AT1G47056.1	F-box protein At1g47056	4.1	1.97E-05
AT4G35320.1	AT4g35320/F23E12_120	4.1	2.91E-04
AT1G31850.3	Probable methyltransferase PMT20 (EC 2.1.1.-)	3.7	9.55E-08
AT1G32900.1	Granule-bound starch synthase 1,	3.6	1.26E-07
AT4G15640.1	Adenylyl cyclase (At4g15640)	2.9	1.17E-03
AT1G21770.1	Acyl-CoA N-acyltransferases (NAT) superfamily protein	2.9	1.34E-07
AT1G64910.1	UDP-glycosyltransferase 79B10 (EC 2.4.1.-)	2.9	4.13E-03
AT1G75170.1	Sec14p-like phosphatidylinositol transfer family protein	2.9	1.42E-02
AT4G20760.1	AT4g20760/F21C20_110 (NAD(P)-binding Rossmann protein	2.8	2.46E-10
AT5G19250.1	Uncharacterized GPI-anchored protein At5g19250	2.8	2.04E-03
AT1G28140.1	F3H9.20 protein (Integral membrane family protein)	2.8	2.04E-03
AT1G03140.1	Pre-mRNA-splicing factor 18	2.7	7.50E-03
AT5G62980.1	Dihydroneopterin aldolase 2 (DHNA2)	2.7	1.46E-04
AT2G18700.1	Probable alpha,alpha-trehalose-phosphate synthase	2.6	4.28E-02
AT5G56340.1	RING-type E3 ubiquitin transferase	2.5	4.16E-02
AT2G04570.1	GDSL esterase/lipase At2g04570	2.4	1.05E-03
AT3G52110.1	At3g52110 (Interferon-activable protein)	2.4	8.04E-03
AT5G48375.1	Putative myrosinase 3 (Sinigrinase 3)	2.3	2.36E-04
AT5G58640.1	AT5g58640/mzn1_90 (Selenoprotein, Rdx type)	2.3	2.32E-06
AT3G07090.1	PPPDE putative thiol peptidase family protein	2.2	3.72E-02

Accession	Protein names	Fold-change	p-value
AT3G17930.1	AT3g17930/MEB5_15	2.2	8.30E-04
AT1G55980.1	AT1G56000 protein (At1g55980/T6H22.19)	2.2	2.16E-04
AT2G17972.1	Expressed protein (Transmembrane protein)	2.2	6.12E-03
AT2G06990.1	DExH-box ATP-dependent RNA helicase DExH10	2.1	9.92E-03
AT3G57340.2	AT3G57340 protein	2.0	4.99E-02
AT1G50000.1	16S rRNA (uracil(1498)-N(3))-methyltransferase	2.0	2.87E-02
AT1G07230.1	Non-specific phospholipase C1	1.9	4.81E-02
AT2G25310.1	ER membrane protein complex subunit-like protein (DUF2012)	1.9	3.76E-03
AT2G17705.1	Methionine-S-oxide reductase	1.9	4.75E-03
AT5G24165.1	At5g24165 (Uncharacterized protein At5g24165)	1.9	1.59E-05
AT5G15970.1	Stress-induced protein KIN2 (Cold-induced protein COR6.6)	1.9	1.34E-05
AT3G06610.1	DNA-binding enhancer protein-like protein	1.8	1.12E-03
AT3G07180.1	GPI transamidase component PIG-S-like protein	1.8	8.59E-03
AT2G45930.1	Expressed protein	1.7	3.18E-03
AT1G60000.1	AT1G60000 protein (Nucleic acid-binding protein, putative)	1.7	8.75E-03
AT4G34150.1	AT4g34150/F28A23_90 (At4g34150)	1.7	2.41E-04
AT5G47890.1	NADH dehydrogenase [ubiquinone] 1 alpha subcomplex subunit 2	1.7	1.32E-03
AT4G21090.1	Adrenodoxin-like protein 2	1.7	2.93E-02
AT5G11580.1	AT5g11580/F15N18_170	1.7	3.05E-02
AT5G52800.3	DNA primase	1.7	5.16E-04
AT2G27730.1	Uncharacterized protein At2g27730, mitochondrial	1.6	4.17E-05
AT2G28630.1	3-ketoacyl-CoA synthase 12 (KCS-12)	1.6	3.55E-02
AT5G47030.1	ATP synthase subunit delta'	1.6	2.16E-04
AT5G51545.1	Protein LOW PSII ACCUMULATION 2, chloroplastic	1.5	3.69E-02
AT1G54630.1*	Carrier of the growing fatty acid chain in fatty acid biosynthesis.	2.5	1.66E-02
AT3G54670.3	Structural maintenance of chromosomes protein 1	6.2	1.42E-07

Supplementary Table S2. List of proteins up-regulated in the leaf epidermis at 24 hpi. Asterisk (*) indicates proteins up-regulated in the leaf epidermis and in the whole leaf.

Accession	Protein names	Fold-change	p-value
**6 Proteins related to stomatal closure and epidermal cell fate**			
AT2G46410.1	Transcription factor CPC (Protein CAPRICE)	25.01	9.55E-07
AT3G10220.1	Tubulin-folding cofactor B (AtTFCB)	20.82	2.01E-04
AT4G20140.1	LRR receptor-like serine/threonine-protein kinase GSO1	8.50	4.74E-15
AT2G05100.1	Chlorophyll a-b binding protein 2.1, chloroplastic	5.03	4.62E-05
AT5G25980.2	Myrosinase 2 (Thioglucosidase 2)	2.00	7.84E-03
AT3G25520.1	Protein ASYMMETRIC LEAVES1/2 ENHANCER 6	1.84	2.57E-02
**8 Receptor proteins**			
AT1G48480.1	Probable inactive receptor kinase At1g48480	65.67	2.16E-16
AT1G72480.1*	Lung seven transmembrane receptor family protein	40.16	2.16E-16
AT4G33430.2	BRASSINOSTEROID INSENSITIVE1-associated receptor kinase 1	23.02	1.50E-07
AT1G70520.1	Cysteine-rich receptor-like protein kinase 2 (Cysteine-rich RLK2)	10.14	4.40E-04
AT1G52290.1	Proline-rich receptor-like protein kinase (AtPERK15)	6.88	3.47E-02
AT3G47110.1	Putative receptor-like protein kinase At3g47110	3.09	2.68E-06
AT5G61480.1	Leucine-rich repeat receptor-like protein kinase TDR	5.56	3.60E-03
AT5G38470.1	Ubiquitin receptor RAD23d (AtRAD23d)	1.82	4.91E-02
**13 Proteins related to cell wall organization**			
AT5G17420.1	Cellulose synthase A catalytic subunit 7 (AtIRX3)	100	2.16E-16
AT5G49215.1	Pectin lyase-like superfamily protein	100	2.16E-16
AT1G75500.2	Protein WALLS ARE THIN 1	34.30	1.40E-09
AT1G69530.3	Expansin-A1 (AtEXPA1) (Alpha-expansin-1) (At-EXP1)	31.85	2.16E-16
AT1G32180.1	Putative cellulose synthase-like protein D6 (AtCslD6)	3.83	5.23E-03
AT1G55120.1	Beta-fructofuranosidase, (Sucrose hydrolase 3)	3.60	2.02E-02
AT3G09410.1	Pectin acetylesterase 5	3.54	2.18E-02
AT2G40610.1	Expansin-A8 (AtEXPA8)	2.61	3.89E-02
AT3G45970.1	Expansin-like A1 (At-EXPL1)	2.37	2.05E-03
AT3G14310.1	Pectinesterase/pectinesterase inhibitor 3	2.30	1.53E-03
AT5G20950.2	Beta-D-glucan exohydrolase-like protein	2.03	1.50E-02
AT2G04780.1	Fasciclin-like arabinogalactan protein 7	1.94	1.95E-02
AT3G62110.1	AT3g62110 (Pectin lyase-like superfamily protein)	1.84	3.06E-02
**24 Transmembrane proteins (transport-related)**			
AT4G32160.1	PX domain-containing protein EREL1 (Protein EREX-like 1)	100	2.16E-16
AT4G14950.1	Vacuole membrane protein KMS1	100	2.16E-16
AT1G01620.1	Aquaporin PIP1-3 (AtPIP1;3)	100	2.16E-16
AT1G27330.1	At1g27330 (Ribosome associated membrane protein RAMP4)	63.23	2.16E-16
AT4G13220.1	At4g13220 (Transmembrane protein)	39.01	9.77E-14
AT5G64410.1	Oligopeptide transporter 4 (AtOPT4)	47.81	7.39E-11
AT3G09300.1	Oxysterol-binding protein-related protein 3B	36.22	4.10E-10
AT5G40660.1	AT5g40660/MNF13_180 (ATP12 protein-like protein)	31.09	2.16E-16
AT1G14670.1	Transmembrane 9 superfamily member 2	21.06	8.75E-07

Accession	Protein names	Fold-change	p-value
AT1G32210.1	Dolichyl-diphosphooligosaccharide DAD1 (AtDAD1)	15.09	3.12E-05
AT2G43130.1	Ras-related protein RABA5c (AtRABA5c)	11.15	6.25E-09
AT5G62890.1	Nucleobase-ascorbate transporter 6 (AtNAT6)	10.06	8.48E-04
AT2G45000.1	Nuclear pore complex protein NUP62 (Nucleoporin 62)	8.65	1.75E-03
AT5G38980.1	At5g38980 (Transmembrane protein)	8.91	1.98E-06
AT3G22290.1	Endoplasmic reticulum vesicle transporter protein	7.60	1.76E-05
AT5G63040.1	Emb|CAB81845.1 (Transmembrane protein)	7.45	4.35E-03
AT2G47490.1	Nicotinamide adenine dinucleotide transporter 1, (AtNDT1)	6.86	4.59E-03
AT3G53610.2	Ras-related protein RABE1a (AtRABE1a) (AtRab8B)	6.58	9.71E-04
AT2G46800.2	Metal tolerance protein 1 (AtMTP1)	5.91	1.86E-06
AT5G03555.1	Purine-uracil permease NCS1 (AtNCS1)	3.31	4.49E-06
AT5G36100.1	Gb|AAD26867.1 (Transmembrane protein)	3.54	1.01E-02
AT5G45750.1*	Ras-related protein RABA1c (AtRABA1c)	6.94	1.28E-02
AT1G69295.1	Plasmodesmata callose-binding protein 4 (AtPDCB4)	4.75	9.84E-04
AT5G20650.1	Copper transporter 5 (AtCOPT5)	2.17	5.91E-03
**11 Defense response proteins**			
AT4G10500.1	Protein DMR6-LIKE OXYGENASE 1	100	2.16E-16
AT1G03150.1	N-terminal acetyltransferase B complex catalytic subunit NAA20	100	2.16E-16
AT5G16840.2	Binding partner of ACD11 1	61.08	2.16E-16
AT1G49780.1	U-box domain-containing protein 26(Plant U-box protein 26)	59.62	2.16E-16
AT3G22600.1	Non-specific lipid transfer protein GPI-anchored 5 (AtLTPG-5)	59.17	1.56E-13
AT3G46730.1	Putative disease resistance RPP13-like protein 3	9.92	2.90E-05
AT4G09430.1	Disease resistance protein (TIR-NBS-LRR class)	6.81	2.18E-06
AT3G19190.1	Autophagy-related protein 2 (AtAPG2)	5.01	1.53E-03
AT2G03440.1	Nodulin-related protein 1 (AtNRP1)	2.21	2.99E-03
AT2G26010.1	Plant defensin 1.3	5.61	1.26E-02
AT2G42530.1	Protein COLD-REGULATED 15B, chloroplastic (AtCOR15B)	1.83	4.14E-02
**20 Proteins related to abiotic stress response and detoxification**			
AT3G23560.1	Protein DETOXIFICATION 19 (AtDTX19)	100	2.16E-16
AT3G51610.1	ATP-dependent helicase/deoxyribonuclease subunit B	100	2.16E-16
AT2G41100.3	Calmodulin-like 12 (Touch-induced calmodulin-related protein 3)	100	2.16E-16
AT2G30170.1	Probable protein phosphatase 2C 26 (AtPP2C26)	100	2.16E-16
AT2G04450.1	Nudix hydrolase 6 (AtNUDT6)	100	2.16E-16
AT5G35735.1	Cytochrome b561 and DOMON domain-containing protein	32.14	4.74E-15
AT3G19490.1	Sodium/proton antiporter 1 (AtNHD1) (Na(+)/H(+) antiporter 1)	35.05	2.16E-16
AT5G18040.1	Protein HEAT-INDUCED TAS1 TARGET 2	30.84	6.43E-10
AT1G54410.1*	Dehydrin HIRD11	23.11	2.16E-16
AT3G16670.1	AT3g16670/MGL6_12	14.26	2.16E-16
AT5G66400.1	Dehydrin Rab18	5.04	4.50E-13
AT1G76180.1	Dehydrin ERD14	2.07	4.31E-03
AT2G39460.1	60S ribosomal protein L23a-1 (AtRPL23A-1)	2.99	9.48E-06
AT1G52690.1	Late embryogenesis abundant protein 7	2.87	2.01E-05
AT5G10010.1	Protein HEAT INTOLERANT 4	2.07	2.58E-02

Accession	Protein names	Fold-change	p-value
AT1G20450.1	Dehydrin ERD10 (Low-temperature-induced protein LTI45)	1.93	1.40E-02
AT5G23750.1	Remorin 1.4 (AtREM1.4) (Remorin group 1 member 4)	1.90	1.58E-02
AT3G13100.1	Multidrug resistance-associated protein 7	3.39	3.93E-05
AT5G15970.1	Stress-induced protein KIN2 (Cold-induced protein COR6.6)	2.67	1.21E-04
AT5G52300.1	Low-temperature-induced (Desiccation-responsive protein 29B)	1.77	4.43E-02
**22 Transcription factors and transcription related-proteins**			
AT1G17440.2	Transcription initiation factor TFIID subunit 12b	100	2.16E-16
AT2G38560.1	Transcription elongation factor TFIIS	100	2.16E-16
AT3G47610.1	Transcription regulator/ zinc ion binding protein	100	2.16E-16
AT4G16150.1	Calmodulin-binding transcription activator 5	100	2.16E-16
AT5G42060.1	Mediator-associated protein 3	100	2.16E-16
AT5G51110.1	Probable pterin-4-alpha-carbinolamine dehydratase	100	2.16E-16
AT5G50320.1	Elongator complex protein 3 (AtELP3) -LEAVES-TWO1	100	2.16E-16
AT5G27720.1	Sm-like protein LSM4 (AtLSM4)	100	2.16E-16
AT2G46310.1*	Ethylene-responsive transcription factor CRF5	42.56	5.69E-10
AT4G31440.1	Transcriptional regulator of RNA polII, SAGA, subunit	40.24	2.16E-16
AT5G61030.1	Glycine-rich RNA-binding protein 3, mitochondrial	31.24	1.17E-08
AT3G06930.2	Probable histone-arginine methyltransferase 1.3 (AtPRMT13)	19.27	6.48E-13
AT5G10960.1	Probable CCR4-associated factor 1 homolog 10	8.57	2.12E-02
AT1G62110.1	Mitochondrial transcription termination factor family protein	4.90	1.54E-03
AT3G02550.1	LOB (ASYMMETRIC LEAVES 2-like protein 38)	4.77	1.01E-05
AT3G15810.1	Protein LURP-one-related 12	3.91	1.41E-02
AT5G64420.1	DNA polymerase V family	3.81	1.86E-02
AT4G26000.1	RNA-binding KH domain-containing protein PEPPER	3.12	2.46E-02
AT4G10920.1	RNA polymerase II transcriptional coactivator KELP	3.09	2.35E-02
AT2G03870.2	Sm-like protein LSM7 (AtLSM7)	2.07	3.78E-02
AT4G40030.2	Histone H3.3 (Histone H3.2)	48.75	2.16E-16
AT1G10200.1	LIM domain-containing protein WLIM1 (AtWLIM1)	2.93	4.05E-02
**20 Ribosomal proteins**			
AT1G68590.1	30S ribosomal protein 3-1	8.01	7.93E-10
ATCG00065.1	30S ribosomal protein S12	48.19	2.16E-16
AT1G79850.1*	30S ribosomal protein S17,	26.89	2.16E-16
AT2G38140.1	30S ribosomal protein S31	3.42	5.64E-04
AT3G48930.1	40S ribosomal protein S11-1	3.00	6.04E-06
AT5G23740.1	40S ribosomal protein S11-3	1.89	3.95E-02
AT1G07770.1	40S ribosomal protein S15a-1	1.86	1.73E-02
AT3G53870.1	40S ribosomal protein S3-2	2.12	2.73E-02
AT5G20290.1	40S ribosomal protein S8-1	1.80	4.95E-02
AT5G47930.1*	40S ribosomal protein S27-3	41.90	2.16E-16
AT3G43980.1	40S ribosomal protein S29	15.51	3.29E-04
AT4G17560.1	50S ribosomal protein L19-1,	2.73	3.79E-02
AT5G65220.1	50S ribosomal protein L29,	2.02	1.06E-02

Accession	Protein names	Fold-change	p-value
AT3G11250.1	60S acidic ribosomal protein P0-3	100	2.16E-16
AT1G43170.6	60S ribosomal protein L3-	2.06	4.92E-03
AT4G18100.1	60S ribosomal protein L32-1	1.79	4.50E-02
AT1G41880.1	60S ribosomal protein L35a-2	14.43	2.16E-16
AT3G53740.2	60S ribosomal protein L36-2	7.84	2.89E-03
AT1G15250.1*	60S ribosomal protein L37-1	9.06	4.07E-13
AT5G02870.1	60S ribosomal protein L4-2 (L1)	1.81	3.27E-02
**6 Translation initiation factors**			
AT1G66070.2	Eukaryotic translation initiation factor 3 subunit J (eIF3j)	11.90	4.14E-09
AT5G35680.3	Eukaryotic translation initiation factor 4C	5.23	5.12E-04
AT4G18040.1	Eukaryotic translation initiation factor 4E-1 (mRNA cap-binding)	25.67	5.01E-10
AT1G76810.1	Eukaryotic translation initiation factor 5B	2.11	1.87E-02
AT1G54290.1	Protein translation factor SUI1 homolog 2	29.32	8.11E-13
AT2G39795.1	Uncharacterized protein At2g39795, mitochondrial	8.76	2.46E-03
**4 Proteinases**			
AT5G23210.1	Serine carboxypeptidase-like 34 (EC 3.4.16.-)	7.03	1.01E-05
AT5G62140.1	ATP-dependent Clp protease ATP-binding subunit (At5g62140)	3.35	5.10E-07
AT4G30910.1	Leucine aminopeptidase 3 (Proline aminopeptidase 3)	2.78	1.10E-02
AT2G44210.2	At2g44210/F4I1.2 (Carboxyl-terminal peptidase (DUF239))	2.69	4.91E-02
**2 Protein inhibitors**			
AT1G55260.1	Bifunctional inhibitor/lipid-transfer protein	2.03	6.04E-03
AT4G16500.1	Cysteine proteinase inhibitor 4 (AtCYS-4)	1.99	1.74E-02
**15 Proteins related to plant growth and development**			
AT4G26410.1	RGS1-HXK1-interacting protein 1	100	2.16E-16
AT1G67290.1	Aldehyde oxidase GLOX1 (Glyoxal oxidase 1)	100	2.16E-16
AT1G65470.1	Chromatin assembly factor 1 subunit FAS1	100	2.16E-16
AT1G10522.1	Protein PLASTID REDOX INSENSITIVE 2, chloroplastic	69.97	6.06E-14
AT4G10760.1	N6-adenosine-methyltransferase MT-A70-like	55.02	2.16E-16
AT1G75750.1	Gibberellin-regulated protein 1 (GAST1 protein homolog 1)	50.74	2.16E-16
AT3G10405.1	At3g10405 (Vacuolar acid trehalase)	27.02	9.52E-13
AT1G43620.1	Sterol 3-beta-glucosyltransferase UGT80B1	28.349	2.21E-08
AT3G11450.1	DnaJ and Myb-like DNA-binding domain-containing protein	26.56	4.19E-05
AT2G46720.1	3-ketoacyl-CoA synthase 13 (KCS-13)	7.315	4.51E-06
AT1G71720.1	Protein PIGMENT DEFECTIVE 338	6.64	5.90E-06
AT3G02480.1	Late embryogenesis abundant protein (LEA)	5.98	4.67E-14
AT5G48840.1	Pantoate--beta-alanine ligase (Pantoate-activating enzyme)	3.35	3.26E-02
AT3G17520.1*	Late embryogenesis abundant protein (LEA) family protein	2.07	3.04E-03
AT1G32415.1	Pentatricopeptide repeat-containing protein At1g32415	2.17	1.96E-02
**127 Other proteins**			
AT2G04845.1	Acyl-CoA N-acyltransferases (NAT) superfamily protein	100	2.16E-16

Accession	Protein names	Fold-change	p-value
AT1G15410.1	Aspartate-glutamate racemase family (F9L1.36 protein)	100	2.16E-16
AT1G14380.3	At1g14380 (IQ-domain 28)	100	2.16E-16
AT1G16960.1	F6I1.3 protein (Ubiquitin domain-containing protein)	100	2.16E-16
AT1G18660.4	At1g18660 (Expressed protein)	100	2.16E-16
AT1G22520.2	At1g22520 (MICOS complex subunit Mic10-like protein	100	2.16E-16
AT1G36320.1	At1g36320/F7F23_4	100	2.16E-16
AT5G22340.2	At5g22340 (NF-kappa-B inhibitor-like protein)	100	2.16E-16
AT5G45040.1	Cytochrome c6, chloroplastic (Cytochrome c-552) (Atc6)	100	2.16E-16
AT1G16410.1	Dihomomethionine N-hydroxylase (Cytochrome P450 79F1)	100	2.16E-16
AT2G21440.1	Expressed protein (RNA-binding (RRM/RBD/RNP motifs)	100	2.16E-16
AT1G31150.1	F28K20.8 protein (K-box region protein (DUF1985))	100	2.16E-16
AT2G26160.1	F-box protein At2g26160	100	2.16E-16
AT2G26030.1	F-box/FBD/LRR-repeat protein At2g26030	100	2.16E-16
AT2G37050.3	Leucine-rich repeat protein kinase family protein	100	2.16E-16
AT4G26965.1	NADH dehydrogenase 1 alpha subcomplex subunit 12	100	2.16E-16
AT5G64080.1	Non-specific lipid transfer protein GPI-anchored 31 (AtLTPG-31)	100	2.16E-16
AT3G04760.1	Pentatricopeptide repeat-containing protein	100	2.16E-16
AT4G19830.1	Peptidyl-prolyl cis-trans isomerase FKBP17-1	100	2.16E-16
AT3G50520.1	Phosphoglycerate mutase-like protein 4	100	2.16E-16
AT2G43235.1	Phosphoribosylformylglycinamidine synthase	100	2.16E-16
AT4G22150.1	Plant UBX domain-containing protein 3 (PUX3)	100	2.16E-16
AT5G36950.1	Protease Do-like 10, mitochondrial	100	2.16E-16
AT5G50000.1	Protein kinase (Protein kinase superfamily protein)	100	2.16E-16
AT4G24680.1	Protein MODIFIER OF SNC1 1 (MODIFIER OF snc1, 1)	100	2.16E-16
AT3G62240.1	RING-type E3 ubiquitin transferase	100	2.16E-16
AT2G07599.2	Uncharacterized protein	100	2.16E-16
AT1G19520.1	Pentatricopeptide repeat-containing protein At1g19525	79.66	2.16E-16
AT5G59470.1	Mannose-P-dolichol utilization defect 1 protein homolog 1	70.35	2.16E-16
AT1G66820.1	Glycine-rich protein	66.84	2.16E-16
AT1G70430.1	Protein kinase superfamily protein	57.87	2.16E-16
AT5G24165.1	Uncharacterized protein At5g24165	46.96	2.16E-16
AT2G45930.1	Expressed protein	40.52	2.16E-16
AT3G61960.1	Serine/threonine-protein kinase ATG1a (AtAPG1a)	38.92	4.92E-08
AT1G22840.1	Cytochrome c-1 (Cytochrome c At1g22840)	38.35	3.57E-10
AT4G26720.1	Serine/threonine-protein phosphatase PP-X isozyme 1	38.19	2.14E-10
AT1G51650.1	ATP synthase subunit epsilon, (ATPase subunit epsilon)	37.94	2.96E-10
AT3G62810.1*	At3g62810 (Complex 1 family protein / LVR family protein)	37.75	1.67E-09
AT4G13180.1	AT4g13180/F17N18_70 (NAD(P)-binding Rossmann-fold	37.32	2.16E-16
AT3G28460.1	Methyltransferase	37.24	1.12E-09
AT1G04190.1*	Tetratricopeptide repeat (TPR)-like superfamily protein	32.91	1.41E-14
AT4G28660.2	Photosystem II reaction center PSB28 protein	30.42	6.89E-10
AT1G59760.1	DExH-box ATP-dependent RNA helicase DExH9	29.27	2.16E-16
AT5G04850.2	Vacuolar protein sorting-associated protein 60.2	29.19	4.55E-08
AT5G52100.1	Dihydrodipicolinate reductase-like protein CRR1	25.63	1.47E-13
AT4G08960.1	Serine/threonine-protein phosphatase 2A activator	24.94	1.62E-07

Accession	Protein names	Fold-change	p-value
AT1G76955.1	At1g76955 (Expressed protein)	23.59	4.10E-05
AT1G20816.1	Outer envelope pore protein 21A,	20.83	1.89E-06
AT5G16010.1	3-oxo-5-alpha-steroid 4-dehydrogenase family protein	20.18	2.16E-16
AT3G59810.1	Sm-like protein LSM6A (AtLSM6A)	20.06	2.16E-16
AT3G05520.2	F-actin-capping protein subunit alpha (CapZ alpha)	19.84	1.40E-04
AT5G62980.1	Dihydroneopterin aldolase 2 (DHNA2)	18.96	1.82E-06
AT4G08160.1	Glycosyl hydrolase family 10 protein	17.27	1.62E-10
AT4G32920.1	Glycine-rich protein	15.03	2.22E-04
AT3G59890.1	4-hydroxy-tetrahydrodipicolinate reductase 2	14.97	8.08E-05
AT1G73400.1	Pentatricopeptide repeat-containing protein At1g73400,	14.55	5.42E-11
AT1G74390.1	Protein NEN3	14.4	1.79E-10
AT2G23670.1	At2g23670/F26B6.32 (Homolog of Synechocystis YCF37)	13.95	2.16E-16
AT4G37270.1	Probable cadmium/zinc-transporting ATPase HMA1,	13.76	5.99E-05
AT4G17030.1	Expansin-like B1 (At-EXPR1) (Ath-ExpBeta-3.1)	13.24	2.95E-03
AT2G14750.1	Adenylyl-sulfate kinase 1, (APS kinase 1)	12.27	9.90E-10
AT2G22795.1	Uncharacterized protein	12.23	4.77E-04
AT2G20690.1	At2g20690/F5H14.34 (Lumazine-binding family protein)	11.39	4.74E-15
AT1G03650.1	Acyl-CoA N-acyltransferases (NAT) superfamily protein	10.91	2.30E-08
AT3G48140.1*	AT3G48140 protein (HVB12D homologue)	10.69	3.71E-04
AT4G29090.1	Putative reverse transcriptase/RNA-dependent DNA polymerase	10.53	2.00E-07
AT2G44620.1	Acyl carrier protein 1	10.14	6.60E-04
AT3G48560.1*	Protein CHLORSULFURON RESISTANT 1	10.13	2.14E-13
AT1G01600.1	Cytochrome P450 86A4	10.05	1.10E-04
AT2G46170.1*	Reticulon-like protein B5 (AtRTNLB5)	9.74	2.06E-08
AT1G05270.1	At1g05270 (TraB family protein)	9.63	3.04E-04
AT1G60000.1	AT1G60000 protein (Nucleic acid-binding protein, putative)	9.53	1.43E-07
AT3G52920.2	RAB6-interacting golgin	8.78	3.95E-07
AT2G35120.1	Glycine cleavage system H protein 2, mitochondrial	8.26	5.00E-07
AT2G23910.1	At2g23910 (NAD(P)-binding Rossmann-fold proteins	8.24	1.64E-03
AT1G16560.2	Post-GPI attachment to proteins factor 3	7.88	1.29E-02
AT1G14300.2	Protein HGH1 homolog	7.66	3.49E-02
AT3G19000.1	2-oxoglutarate (2OG) and Fe(II)-dependent oxygenase protein	7.13	5.48E-06
AT2G47640.1	Small nuclear ribonucleoprotein Sm D2 (Sm-D2)	6.94	1.25E-07
AT2G21860.1	At2g21860/F7D8.18 (Violaxanthin de-epoxidase-like protein)	6.66	4.77E-03
AT3G51520.1*	Diacylglycerol O-acyltransferase 2 (AtDGAT2)	6.59	5.66E-03
AT1G03100.1	Pentatricopeptide repeat-containing protein At1g03100	6.27	2.16E-16
AT3G07090.1	PPPDE putative thiol peptidase family protein (T1B9.26 protein)	6.09	2.16E-04
AT5G06180.1	AT5G06180 protein (Fission ELM1-like protein (DUF1022)	5.8	3.73E-02
AT3G23760.1	Transferring glycosyl group transferase	5.68	3.12E-05
AT2G40480.1	WEB family protein At2g40480	5.63	1.13E-04
AT5G47030.1*	ATP synthase subunit delta', (F-ATPase delta' subunit)	5.4	3.91E-05
AT1G04280.1	Calmodulin calcium-dependent NAD kinase	5.17	3.47E-02
AT1G35420.2	Alpha/beta-Hydrolases superfamily protein	5.12	1.52E-05
AT4G33625.1	At4g33625 (Vacuole protein)	5.08	5.13E-06
AT2G18040.1	Peptidyl-prolyl cis-trans isomerase Pin1 (PPIase Pin1)	5.01	9.87E-07

Accession	Protein names	Fold-change	p-value
AT2G28630.1	3-ketoacyl-CoA synthase 12 (KCS-12)	5	8.52E-03
AT5G52800.3	DNA primase	4.96	3.08E-12
AT1G36280.1	Adenylosuccinate lyase (ASL) (Adenylosuccinase)	4.86	5.04E-04
AT2G43810.1	Sm-like protein LSM36B (AtLSM6B)	4.66	2.55E-03
AT2G20560.1	At2g20560/T13C7.15 (DNAJ heat shock family protein)	4.21	7.69E-03
AT5G48375.1	Putative myrosinase 3 (EC 3.2.1.147) (Beta-glucosidase 39)	4.09	1.02E-02
AT1G50520.1	Cytochrome P450, family 705, subfamily A, polypeptide 27	4.08	8.14E-03
AT3G16390.1	Nitrile-specifier protein 3 (AtNSP3) (Jacalin-related lectin 27)	4.03	2.17E-03
AT3G18940.1	Proteasome assembly chaperone 2	3.69	1.20E-08
AT3G17930.1	AT3g17930/MEB5_15 (Transmembrane protein)	3.64	2.99E-03
AT1G60550.1	1,4-dihydroxy-2-naphthoyl-CoA synthase, peroxisomal (DHNS)	3.5	2.60E-02
AT2G17972.1	Expressed protein (Transmembrane protein)	3.47	1.58E-02
AT4G21090.1	Adrenodoxin-like protein 2 (AtMFDX2)	3.47	4.00E-03
AT3G49430.1	Serine/arginine-rich splicing factor SR34A (At-SR34A)	3.13	4.83E-02
AT5G56090.1	Cytochrome c oxidase assembly protein COX15	3.04	2.37E-03
AT4G27585.1	SPFH/Band 7/PHB domain-containing membrane-associated	2.94	2.29E-03
ATCG00710.1	Photosystem II reaction center protein H (PSII-H)	2.68	2.93E-05
AT3G22480.2	Probable prefoldin subunit 2	2.63	1.89E-02
AT3G52340.2	Probable sucrose-phosphatase 3b (AtSPP3b)	2.62	2.15E-02
ATCG00730.1	Cytochrome b6-f complex subunit 4 (17 kDa polypeptide)	2.48	2.39E-04
AT5G28750.1	Sec-independent protein translocase protein TATA	2.47	3.54E-02
AT4G28025.1	Uncharacterized protein At4g28025	2.43	2.64E-02
AT4G01150.1	Protein CURVATURE THYLAKOID 1A, chloroplastic	2.23	1.35E-03
AT1G14980.1	10 kDa chaperonin, mitochondrial (Chaperonin 10) (CPN10)	2.22	2.68E-03
AT3G21055.1	Photosystem II 5 kDa protein, chloroplastic (PSII-T)	2.12	6.99E-03
AT1G73110.1	At1g73110/F3N23_39	2.1	2.77E-02
AT5G65780.1	Branched-chain-amino-acid aminotransferase 5, (Atbcat-5)	2.05	4.38E-02
AT5G39570.1*	Uncharacterized protein At5g39570	1.97	1.43E-02
AT3G63160.1	AT3g63160/F16M2_10 (At3g63160) (Outer envelope membrane)	1.93	1.86E-02
AT1G33811.1	GDSL esterase/lipase At1g33811	1.88	4.76E-02
AT1G10590.3	Nucleic acid-binding, OB-fold-like protein (T10O24.24)	1.88	4.69E-02
AT1G15270.1	F9L1.21 protein (Translation machinery associated TMA7)	1.85	4.87E-02
AT1G76100.1	Plastocyanin minor isoform, chloroplastic	1.82	2.92E-02
AT2G24420.2	AT2G24420 protein (At2g24420/T28I24.15)	1.82	4.03E-02
AT1G65020.1	At1g65020 (Plasma protein)	1.77	4.47E-02
AT3G26740.1	Light-regulated protein 1 (Protein CCR-LIKE, chloroplastic)	1.76	3.50E-02

Supplementary Table S3. List of fungal proteins expressed on the leaf surface. BP: biological process; MF: molecular function

Uniprot ID	Protein name	SignalP	EffectorP	Ontology
Specifically identified proteins at 24 hpi samples				
I1REV2	Uncharacterized protein	Secretory	Non-effector	Hydrolase activity, acting on carbon-nitrogen (but not peptide) bonds, in linear amidines (MF); metal ion binding (MF)
I1RPJ8	Uncharacterized protein	Secretory	Non-effector	Uncharacterized protein
I1RWF4	Cytochrome b5 heme-binding proteins	Others	Non-effector	Heme binding (MF); metal ion binding (MF)
I1RC74	Uncharacterized protein	Others	Non-effector	Catalytic activity (MF); pyridoxal phosphate binding (MF)
I1RDG5	TauD domain-containing protein	Others	Non-effector	TauD domain refers to a protein domain that in many enteric bacteria is used to break down taurine (2-aminoethanesulfonic acid) as a source of sulfur under stress conditions
I1S138	NmrA domain-containing protein	Others	Non-effector	A new protein carrying an NmrA-like domain is required for cell differentiation and development in *Dictyostelium discoideum*
Commonly identified proteins at 4 hpi nd 24 hpi samples				
A0A0E0S530	Uncharacterized protein	Others	Effector	Catalytic activity (MF)
I1RDZ6	Nitroreductase domain-containing protein	Others	Effector	Cellular response to oxidative stress (BP)
I1RMF2	Ypt/Rab-type GTPase Rab7	Others	Effector	Regulates the FgAtg9 for autophagy-dependent development and pathogenicity of *F. graminearum*
I1S287	Uncharacterized protein	Others	Effector	GTPase activity (MF); GTP binding (MF)
I1S361	SGNH_hydro domain-containing protein	Secretory	Effector	SGNH_Hydro domain found in fungal Rhamnogalacturonan acetylesterase which important for *F. graminearum* growth on plant cell wall
A0A098D0Q4	TauD domain-containing protein	Others	Non-effector	Oxidoreductase activity (MF)
A0A098DA88	Fork-head domain-containing protein	Others	Non-effector	Two other Fork-head transcription factors role the fungal growth and pathogenicity
A0A098DJX8	BTB domain-containing protein	Others	Non-effector	Many BTB proteins are transcriptional regulators that are thought to act through the control of chromatin structure.

Uniprot ID	Protein name	SignalP	EffectorP	Ontology
A0A098DP80	Bac_luciferase domain-containing protein	Others	Non-effector	monooxygenase activity (MF); Oxidoreductase activity, acting on paired donors, with incorporation or reduction of molecular oxygen (MF)
A0A098DTE3	GST C-terminal domain-containing protein	Others	Non-effector	Glutathione metabolic process (BP)
A0A098DVM4	Uncharacterized protein	Others	Non-effector	GTPase activity (MF); GTP binding (MF)
A0A0E0RSG8	Aspartate--tRNA ligase	Others	Non-effector	Aspartyl-tRNA aminoacylation (BP); aspartate-tRNA ligase activity (BP); ATP binding (BP)
A0A0E0S8X8	Oxidored_FMN domain-containing protein	Others	Non-effector	FMN binding (MF); oxidoreductase activity (MF)
A0A1C3YJJ0	cobW domain-containing protein	Others	Non-effector	CobW proteins are generally found proximal to the trimeric cobaltochelatase subunit CobN, which is essential for vitamin B12 (cobalamin) biosynthesis
A0A1C3YLC3	Uncharacterized protein	Others	Non-effector	Uncharacterized protein
A0A1C3YM05	Uncharacterized protein	Others	Non-effector	ATPase activity (MF); ATP binding (MF)
I1R9Y9	PCI domain-containing protein	Others	Non-effector	The PCI domain mediates and stabilizes protein-protein interactions within the complexes. The role of the PCI domains is most likely that of a scaffold for the other complex subunits and other binding partners
I1RA13	Acetyl-coenzyme A synthetase	Others	Non-effector	Acetyl-CoA biosynthetic process from acetate (BP)
I1RA52	Complex1_49kDa domain-containing protein	Others	Non-effector	NAD binding (MF); oxidoreductase activity, acting on NAD(P)H (MF); quinone binding (MF)
I1RC52	Uncharacterized protein	Others	Non-effector	Intracellular protein transport (BP)
I1RCZ9	Phospho-2-dehydro-3-deoxyheptonate aldolase	Others	Non-effector	aromatic amino acid family biosynthetic process (BP); chorismate biosynthetic process (BP)
I1RDB1	AAA domain-containing protein	Others	Non-effector	Protein catabolic process (BP)
I1RDN3	Acetyl-coenzyme A synthetase	Others	Non-effector	Acetyl-CoA biosynthetic process from acetate (BP)
I1REA2	CCHC-type domain-containing protein	Others	Non-effector	Zinc finger, nucleic acid binding (MF); zinc ion binding (MF)
I1RJB7	Uncharacterized protein	Others	Non-effector	Isomerase activity (MF)

Uniprot ID	Protein name	SignalP	EffectorP	Ontology
I1RL01	Uncharacterized protein	Others	Non-effector	Uncharacterized protein
I1RMK0	Uncharacterized protein	Others	Non-effector	Uncharacterized protein
I1RN31	Protein kinase domain-containing protein	Others	Non-effector	ATP binding (MF); protein serine/threonine kinase activity (MF)
I1RN43	Protein kinase domain-containing protein	Others	Non-effector	ATP binding (MF); protein kinase activity (MF)
I1RQN9	Mitogen-activated protein kinase	Others	Non-effector	ATP binding (MF); MAP kinase activity (MF)
I1RR84	PKS_ER domain-containing protein	Others	Non-effector	Oxidoreductase activity (MF)
I1RS20	eRF1_1 domain-containing protein	Others	Non-effector	Translation release factor activity (MF)
I1RSU7	Uncharacterized protein	Others	Non-effector	Metal ion binding (MF)
I1RSY9	Aldo_ket_red domain-containing protein	Others	Non-effector	Oxidoreductase activity (MF)
I1RTS4	TauD domain-containing protein	Others	Non-effector	Oxidoreductase activity (MF)
I1RW16	Protein kinase domain-containing protein	Others	Non-effector	ATP binding (MF); protein serine/threonine kinase activity (MF)
I1RW55	Uncharacterized protein	Others	Non-effector	acyl-CoA dehydrogenase activity (MF); flavin adenine dinucleotide binding (MF)
I1RWE8	Methionyl-tRNA synthetase	Others	Non-effector	Methionyl-tRNA aminoacylation (BP)
I1RX54	PH domain-containing protein	Others	Non-effector	Maintenance of protein location in cell cortex (BP)
I1RXF0	Plug_translocon domain-containing protein	Others	Non-effector	Protein transport (BP)
I1RYM9	Drf_GBD domain-containing protein	Others	Non-effector	Actin cytoskeleton organization (BP)
I1RZK4	AAA domain-containing protein	Others	Non-effector	ATPase activity (MF); ATP binding (MF)
I1S0H5	Uncharacterized protein	Others	Non-effector	Hydrolase activity (MF)
I1S1H0	Uncharacterized protein	Others	Non-effector	Monooxygenase activity (MF); oxidoreductase activity, acting on paired donors, with incorporation or reduction of molecular oxygen (MF)
I1S203	NACHT domain-containing protein	Others	Non-effector	The NACHT domain is found in association with other domains, such as the WD repeat, the leucine-rich repeat (LRR) or the BIR repeat

Uniprot ID	Protein name	SignalP	EffectorP	Ontology
Q4IR18	Polynucleotide 5'-hydroxyl-kinase GRC3	Others	Non-effector	Polynucleotide 5'-kinase involved in rRNA processing
V6R5D2	Aldedh domain-containing protein	Others	Non-effector	Oxidoreductase activity, acting on the aldehyde or oxo group of donors, NAD or NADP as acceptor (MF)
V6R7G9	Oxidored_FMN domain-containing protein	Others	Non-effector	FMN binding (MF); oxidoreductase activity (MF)
V6RQ48	AP complex subunit beta	Others	Non-effector	Adaptins are components of the adaptor complexes which link clathrin to receptors in coated vesicles.
I1RFQ1	JAB_MPN domain-containing protein	Others	Non-effector	mRNA splicing, via spliceosome (BP)
I1RZM4	Serine hydroxymethyltransferase	Others	Non-effector	Interconversion of serine and glycine.
A0A0E0SJS1	Uncharacterized protein	Others	Non-effector	GTP binding (MF)
A0A098DJ03	Thioredoxin domain-containing protein	Others	Non-effector	Thiol-specific peroxidase that catalyzes the reduction of hydrogen peroxide and organic hydroperoxides to water and alcohols, respectively.
A0A1C3YHY6	Uncharacterized protein	Others	Non-effector	Uncharacterized protein
I1RFJ1	Thiamine thiazole synthase	Others	Non-effector	Involved in biosynthesis of the thiamine precursor thiazole. May have additional roles in adaptation to various stress conditions and in DNA damage tolerance.
